# Prediction models for self-harm and suicide: a systematic review and critical appraisal

**DOI:** 10.1186/s12916-025-04367-6

**Published:** 2025-10-09

**Authors:** Aida Seyedsalehi, James Bailey, Maya G. T. Ogonah, Thomas R. Fanshawe, Seena Fazel

**Affiliations:** 1 Department of Psychiatry, University of Oxford, Oxford, UK; 2 Research Department of Primary Care and Population Health, University College London, London, UK; 3 Nuffield Department of Primary Care Health Sciences, University of Oxford, Oxford, UK; 4 Oxford Health NHS Foundation Trust, Oxford, UK

**Keywords:** Suicide, Self-harm, Clinical prediction models, Risk prediction, Systematic review

## Abstract

**Background:**

The number of prediction models for self-harm and suicide has grown substantially in recent years. However, their potential role in improving assessment of suicide risk is debated. In this systematic review, we provide an overview and critical appraisal of the predictive performance and methodological quality of prognostic risk models for self-harm and suicide.

**Methods:**

We searched MEDLINE, EMBASE, PsycINFO, CINAHL, and Global Health from inception to 30/11/2021. The search was updated on 25/10/2024 to include new external validations. We included studies describing the development and/or external validation of statistical models for predicting risk of non-fatal self-harm and/or death by suicide. Risk of bias was assessed using the Prediction model Risk Of Bias ASsessment Tool (PROBAST).

**Results:**

We included 91 articles describing the development of 167 models and 29 external validations. Most models predicted risk of self-harm (76 models), followed by suicide (51 models), and the composite outcome of suicide or non-fatal self-harm (40 models). Only 8% of developed models (14/167) were externally validated, and 17% (28/167) were presented in a format enabling validation or use by others. The reported C indices ranged from 0.61 to 0.97 (median 0.82) in development studies and from 0.60 to 0.86 (median 0.81) in external validations. Calibration was assessed for 9% of models (15/167) in development studies and 31% of external validations (9/29). Of these, the OxMIS and Simon models showed adequate discrimination and calibration performance in external validation. All model development studies, and all but two external validations, were at high risk of bias. This was mainly driven by inappropriate or incomplete evaluation of predictive performance (180/196, 92%), insufficient sample sizes (151/196, 77%), inappropriate handling of missing data (129/196, 66%), and not adequately accounting for overfitting and optimism during model development (106/167, 63%).

**Conclusions:**

Despite skepticism about the feasibility and accuracy of self-harm and suicide risk prediction and assessment, we have identified five models with good predictive performance in external validation. Avoidable sources of research waste include an oversupply of unvalidated prediction models addressing similar research questions, and shortcomings in study design, conduct, and statistical analysis. To address these, new research must prioritise methodological rigour and focus on external validation and updating existing models. Complete, transparent, and accurate reporting is essential, with model presentation in a format that enables independent validation.

**Supplementary Information:**

The online version contains supplementary material available at 10.1186/s12916-025-04367-6.

## Background

Suicide risk influences clinical decision-making at all stages of care, from referral and assessment to treatment and discharge from services [1–5]. Prognostic models that combine information on multiple predictors to estimate the future risk of suicide-related outcomes can assist in identifying high-risk individuals and facilitate a stratified medicine approach to prevention [6, 7]. In some medical specialties (e.g. cardiovascular medicine and oncology), prognostic models are routinely used in clinical practice and recommended for risk assessment and therapeutic management in clinical guidelines [8–10]. However, the potential role of prognostic models for suicide risk assessment has been widely debated [1, 2]. For instance, in England, the National Institute for Health and Care Excellence advises against the use of these models to predict the risk of recurrence in individuals who have self-harmed [11], partly based on poor predictive performance and limited clinical utility (although the evidence review on which these recommendations are based is very limited, including only two studies). Instead, some experts have suggested that interventions should be provided to unselected clinical populations (e.g. offering psychological therapy to all individuals presenting to hospital with self-harm). New suicide prevention guidance by NHS England [12] repeats the NICE recommendation regarding current risk prediction tools in self-harm. However, others have argued that the targeted allocation of limited resources in mental health services requires some assessment of risk, which currently relies on clinical subjective judgement alone [1]. By making risk assessment more empirically grounded, consistent, and transparent, prognostic models could enhance clinical decision-making, enabling clinicians to focus on a comprehensive assessment of needs and development of an individualised risk management plan [1].

The number of risk models for suicide-related outcomes has grown substantially in recent years. To evaluate the role of these models in improving assessment of self-harm and suicide risk, high-quality systematic review evidence on their predictive performance and methodological quality is required. While a number of recent systematic reviews have been published (e.g. [13–15]), most have been restricted to models developed using machine learning techniques, focused on inappropriate statistical measures to evaluate predictive performance (e.g. odds ratios), or not sufficiently evaluated the risk of bias and reporting of existing models. To address these limitations, we conducted a systematic review of studies reporting on the development or external validation of a prognostic model for suicide and/or non-fatal self-harm. Our aims were to provide an overview of current models for predicting risk of suicide and self-harm, summarise their predictive performance, and critically appraise their methodological quality and risk of bias.

## Methods

This systematic review is reported according to the Transparent Reporting of multivariable prediction models for Individual Prognosis Or Diagnosis checklist for Systematic Reviews and Meta-Analyses [16] (TRIPOD-SRMA; see Additional file 1). The review protocol was registered on PROSPERO (CRD42022314575). Deviations from the protocol are explained in Additional file 2: Supplementary methods [17–25].

### Searching and selection of articles

We searched MEDLINE, EMBASE, PsycINFO, CINAHL, and Global Health up to 30 November 2021 to identify primary articles reporting on the development and/or validation of prognostic models for suicide and/or self-harm (the search strings are provided in Additional file 2: Supplementary methods). The search was updated on 25 October 2024 to include new external validations of included models (identified in the original search) that had been published since November 2021. The search strategy used for the update was the same as the original search. We screened the reference lists of included articles and previously published systematic reviews to identify additional studies.

We included primary articles if they reported on the development and/or external validation (with or without updating) of one or more multivariable models, based on patient-level data, to estimate the risk of future occurrence of non-fatal self-harm or death by suicide. We defined self-harm as any act of intentional self-injury or self-poisoning, irrespective of motivation and degree of suicidal intent [11]. We did not distinguish between suicide attempts and non-suicidal self-injury, and classified models predicting either of these outcomes as models for non-fatal self-harm. Models predicting a composite endpoint (i.e. non-fatal self-harm or suicide) were also eligible for inclusion. We did not include models predicting suicidal ideation (either as a separate outcome or in combination with self-harm and/or suicide).

There were no restrictions on target population, study setting, intended moment of model use, or prediction horizon (i.e. timeframe for outcome prediction). We excluded predictor finding studies (i.e. studies identifying factors associated with self-harm or suicide) and prediction model impact studies (i.e. using a comparative design to assess the impact of using a prediction model on patient outcomes, clinical decision-making, or cost-effectiveness of care). Prediction modelling studies with cross-sectional designs (e.g. where the outcome was history of self-harm) and ecological studies predicting an aggregate outcome (e.g. trends in the incidence of suicide or self-harm) were also excluded. We did not include any risk assessment scales or checklists; only multivariable models with statistically derived weighting were included. Finally, we excluded studies assessing the accuracy of unassisted clinician judgment.

All references were collated and managed in EPPI-Reviewer Web [26]. AS screened the retrieved records for eligibility, first on title and abstract and then based on full text. A randomly selected 10% of records were independently screened by a second reviewer (MGTO). Discrepancies were resolved by discussion between the two reviewers.

### Data extraction and risk of bias assessment

We classified eligible articles as model development (with or without internal validation), external validation (with or without model updating), or combined model development and external validation. A standardised data extraction and risk of bias assessment form was developed based on the CHecklist for critical Appraisal and data extraction for systematic Reviews of prediction Modelling Studies (CHARMS) [27] and the Prediction model Risk Of Bias ASsessment Tool (PROBAST) [28, 29]. The form was supplemented with items based on a recent review [30], and piloted on a small subset of included articles. The data extraction items (prespecified in the PROSPERO protocol) covered key study characteristics, participants, predictors, outcomes, sample size, statistical analysis methods, model presentation, and predictive performance (including measures of discrimination and calibration and their corresponding standard errors or confidence intervals (CIs), and measures of clinical utility if presented). The full list of data extraction items is available in Additional file 2: Supplementary methods.

Many articles described the development of multiple models, and a few models were investigated in more than one paper (i.e. multiple external validation studies). Unless the authors clearly identified one or more of the reported models as their main proposed model(s), each combination of predictor variables with unique predictor-outcome association estimates and/or a unique estimate of baseline risk/hazard was considered a separate model [31]. See Additional file 2: Supplementary methods for more details on data extraction and risk of bias assessment.

AS extracted the data and assessed the risk of bias for all included articles, with a randomly selected 10% of articles independently assessed by a second reviewer (JB). Any discrepancies were resolved by consensus between the two reviewers and discussion with senior authors (SF and TRF).

### Statistical analyses

We did not perform a quantitative meta-analysis, as this is only applicable when a review aims to summarise the predictive performance of one or more specific prognostic models across multiple external validation studies [32]. The aim of our review was to identify all prognostic models—developed or validated—for suicide death and/or non-fatal self-harm, and we considered meta-analysis not to be of added value for this field synopsis. We therefore provide a descriptive summary of identified models and validations, including participant characteristics, model predictors and outcomes, analysis methods, predictive performance measures, and risk of bias [32]. The unit of analysis for the review was each unique model development and validation [30].

On recommendation of peer review, we conducted post-hoc analyses summarising the discrimination performance of models and external validations stratified by data source (routine data versus prospectively collected data from a cohort study or clinical trial) and by model dimensionality (low- versus high-dimensional). In the methodological literature, settings in which the number of candidate predictors is much larger than the available sample size are often defined as high-dimensional [33–36]. For the purpose of these analyses, we used the number of events per candidate predictor parameter (EPP) as a proxy for dimensionality, classifying models as high-dimensional if EPP < 1 and low-dimensional if EPP ≥ 1.

All analyses were performed in R software (version 4.4.2; The R Foundation for Statistical Computing, Vienna, Austria) [37]. The PRISMA flow diagram was generated using the shiny app by Haddaway et al. [38] (available at https://estech.shinyapps.io/prisma_flowdiagram/).

### Patient and public involvement

No patients or members of the public were involved in developing the research question and outcome measures, in the design and conduct of the study, or in the interpretation of findings. There are no plans to disseminate the study results to relevant patient communities.

## Results

**Fig. 1:**
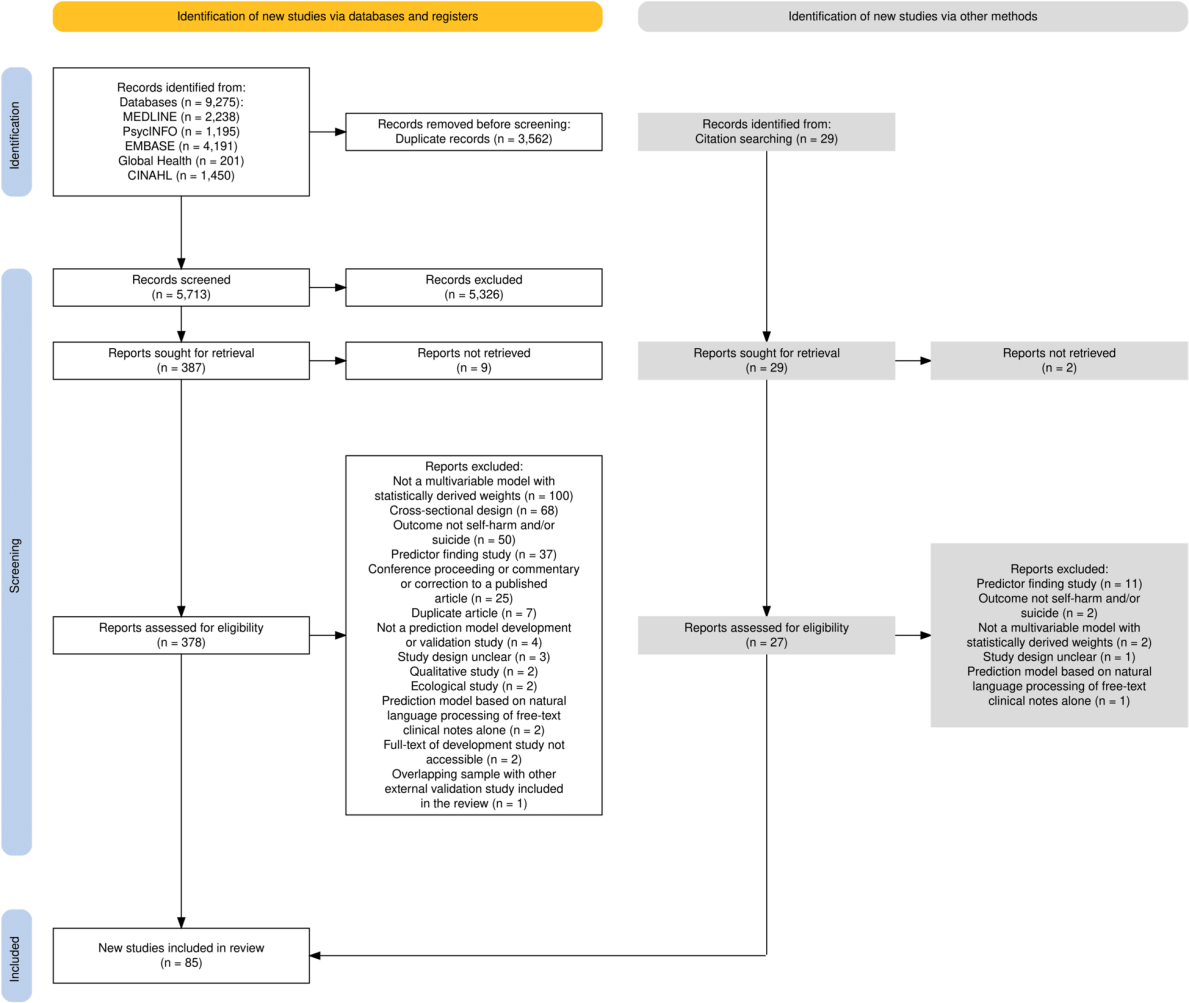
PRISMA (Preferred Reporting Items for Systematic reviews and Meta-analyses) flow diagram of study selection for the original search (conducted on 30 November 2021). The diagram does not include studies from the external validation update

Our original search identified 5, 713 unique records, of which 5, 326 were excluded on title and abstract. The remaining 387 articles were screened on full text, and 75 met eligibility criteria for inclusion. An additional 10 articles were identified from reference lists and previous systematic reviews (Fig. 1). The search update yielded 2, 477 results, of which 6 articles (published since November 2021) reported on an external validation of a model identified by our original search strategy. In total, we included 91 articles. Of these, 82 described the development of prediction models for non-fatal self-harm and/or suicide, and 15 reported on their external validation.

### Overview

The 82 articles described the development of 167 unique models: 76 models for predicting self-harm, 51 models for predicting suicide death, and 40 models for predicting the composite outcome of suicide or non-fatal self-harm. A total of 29 external validations were reported in 15 articles: 13 validations for the composite outcome of suicide or non-fatal self-harm, nine validations for suicide death, and seven for non-fatal self-harm. The majority of the 167 developed models (*n* = 153, 92%) were not externally validated (see Additional file 2: Supplementary results for more details). Additional file 3 provides the full list of reviewed models and validations, with information on their target population, outcome, predictive performance, risk of bias, and whether they were available in a format for validation or use.

### Sample characteristics

Over 60% of newly developed models and external validations were based on data from the USA (*n* = 125, 64%), followed by Canada (*n* = 10, 5%), Denmark (*n* = 10, 5%), Australia (*n* = 8, 4%), and Sweden (*n* = 8, 4%). Only 12 models and validations (6%) originated from outside North America, Europe, and Australia. Almost all external validations (*n* = 26, 90%) used data from the USA (Additional file 2: Table S1).

Most models were developed and externally validated using routine data, such as from electronic health records or administrative databases (72%; Table 1). The most common healthcare setting was mixed clinical settings (36%), followed by outpatient (13%) and inpatient (10%) mental health care, and the most common target population was individuals under mental health services (28%). Mean participant age (where reported) ranged from 14 to 72 years, with most models developed and validated in adults (38%) or in samples composed of all ages (37%). Twenty-five models and validations (14%) were sex-specific (15 restricted to men and 10 to women). Additional study characteristics are presented in Additional file 2: Table S2.

**Table 1:** Characteristics of included models and external validations (*N* = 196)

	*N* (%) *
**Data source**	
Routine data	121 (72%)
Prospective cohort study/clinical trial ^†^	75 (45%)
**Target setting**	
Mixed clinical settings	70 (36%)
Outpatient (mental health)	26 (13%)
Inpatient (mental health)	20 (10%)
Community (general population)	18 (9%)
Emergency department	10 (5%)
Outpatient (general medical)/primary care	9 (5%)
Inpatient (general medical)	6 (3%)
Other	11 (6%)
Unclear	26 (13%)
**Target population**	
Individuals under mental health services (inpatient and outpatient)	55 (28%)
General hospital patients/individuals discharged from general hospital	31 (16%)
General population	30 (15%)
Individuals presenting to the emergency department/hospital with self-harm	17 (9%)
US Army/Navy service members	15 (8%)
Primary care/outpatient general medical care patients with a mental health diagnosis	8 (4%)
Veterans	8 (4%)
Individuals with physical health conditions	3 (2%)
Prisoners	3 (2%)
Other	17 (9%)
Unclear	9 (5%)
**Age group**	
Adults	75 (38%)
Mixed	73 (37%)
Children and adolescents	32 (16%)
Unclear	16 (8%)

^*^ Categories are non-overlapping (i.e. each developed model/external validation only belongs to one category)

^†^ For some models/validations in this category, data from the prospective cohort study or clinical trial was supplemented with routine data (e.g. from administrative databases)

Sample size for model development ranged from 74 to 1,059,184 participants, with a median of 8, 264 (interquartile range (IQR) 1, 021 to 39,028). The number of individuals experiencing suicide-related outcomes ranged from 9 to 13,428 (median 137, IQR 64 to 974). For external validations, number of participants ranged from 325 to 660,659 (median 16,387, IQR 13,761 to 257,680), and number of individuals with outcomes ranged from 18 to 6, 678 (median 806, IQR 46 to 5,672).

### Outcomes

Outcomes were most commonly defined based on International Classification of Disease (ICD) codes (42%) or a combination of different methods (e.g. self-report and ICD codes documented in electronic health records, 15%; see Additional file 2: Table S2 for more details). Prediction horizons varied up to a maximum of 10 years, with most studies predicting risk over a period of 1 year or less. For 17% of models and validations, the prediction horizon was variable across participants (e.g. logistic regression models where participants had different follow-up times), and 10% did not specify a clear prediction horizon. The intended moment of model use for prediction was unclear for 41% of models and validations.

### Analysis methods

The number of candidate predictor parameters (i.e. β terms in the regression equation) considered for inclusion in the model ranged from 9 to over 89,000, with a median of 150 (IQR 27 to 662) parameters. The EPP could be calculated or approximated for 90% of developed models (150/167), and ranged from < 0.01 to 135 (median 1.76, IQR 0.61 to 3.81). The distribution of EPP values is presented in Additional file 2: Fig. S1.

Most models were developed using logistic regression (36%) and tree-based methods (31%; Table 2). Among models that involved variable selection during multivariable modelling (*n* = 92), the most commonly used approaches were the least absolute shrinkage and selection operator (LASSO), forward selection, and elastic net (Table 2). The number of predictor parameters included in the final model ranged from 2 to 8, 071, with a median of 13 (IQR 6 to 29) parameters (see Additional file 2: Supplementary results for details).

**Table 2:** Overview of analysis methods for all developed models (*N* = 167)

	***N* (%)**
**Modelling technique**	
Logistic regression	60 (36%)
Tree-based	51 (31%)
Cox proportional hazards regression	16 (10%)
Other survival model	12 (7%)
Ensemble machine learning method	6 (4%)
Neural network	6 (4%)
Other	16 (10%)
**Variable selection approach**	
Not applicable *	53 (32%)
All predictors forced in model	22 (13%)
LASSO	15 (9%)
Forward selection	13 (8%)
Elastic net	12 (7%)
Backward selection	8 (5%)
Stepwise selection	5 (3%)
Backward (stepwise) selection	3 (2%)
Other	31 (19%)
Unclear	5 (3%)
**Internal validation technique**	
No internal validation	41 (25%)
(Random) split into development and testing datasets	50 (30%)
Cross-validation	39 (23%)
Bootstrapping	26 (16%)
Other	11 (7%)
**Shrinkage/penalisation method**	
Not performed	123 (74%)
LASSO/ridge/elastic net	27 (16%)
Heuristic (uniform) shrinkage	1 (1%)
Other	12 (7%)
Unclear	4 (2%)

^*^ The modelling approach did not result in a discrete set of predictor variables included in the final model (e.g. random forests)

Most developed models (*n* = 126, 75%) were internally validated (Table 2). The most common method of internal validation was a random split into development and validation datasets (*n* = 50), followed by cross-validation (*n* = 39) and bootstrapping (*n* = 26). Shrinkage and penalisation methods were applied during the model development process for 40 models (24%), most commonly using ridge regression, LASSO, or elastic net (Table 2).

Methods to correct for class imbalance (i.e. imbalance in the frequency of individuals with and without the outcome event) [39] were used in the development of 41 models (25%). The studies used different resampling approaches, including random under-sampling, random over-sampling, and the Synthetic Minority Oversampling Technique (SMOTE) [40], all of which artificially inflate the outcome prevalence in the development dataset [41]. Only one model [42] was recalibrated (to the original outcome prevalence) following imbalance correction.

Some developed models (*n* = 26, 16%) and external validations (*n* = 23, 79%) were based on clustered data which could include multiple observations (e.g. clinical visits or hospitalisations) per person. Of these, 24 (49%) only reported sample size information at the visit level (e.g. number of outpatient visits followed by a suicide death within 90 days).

### Model presentation

Overall, only 17% of developed models (28/167) were publicly available in a format for use in clinical practice. For the remaining models, authors did not provide sufficient information to allow calculation of individual risk estimates (Table 3). Eleven models (7%) were available as a tool that could be used to calculate individual risks (in addition to or instead of the model equation), such as a decision tree, a point score system, or an online calculator. Only 20 of the 106 regression-based models were fully presented as a model equation (i.e. including the model intercept/baseline hazard and all regression coefficients; Table 3).

**Table 3:** Availability of models (*N* = 167) in format for use in clinical practice

	***N* (%)**
**Model equation**	
Not applicable (not a regression-based model)	61 (37%)
Partially presented *	44 (26%)
Not presented ^†^	42 (25%)
Fully presented ^‡^	20 (12%)
**Prediction tool**	
Not presented	156 (93%)
Decision tree	4 (2%)
Points score system	3 (2%)
Predictions for specific risk subgroups	2 (1%)
Nomogram	1 (1%)
Online calculator	1 (1%)

^*^ Model equation partially presented (e.g. hazard or odds ratios but no estimate of the intercept or baseline hazard)

^†^ Did not provide any information about the underlying model equation

^‡^ Model equation fully presented (including the model intercept or baseline hazard and all regression coefficients)

### Predictive performance

#### Development and internal validation

In model development studies, a C index was reported for 88% of models (147/167; see Additional file 2: Figs. S2–S4 and Additional file 4). These ranged from 0.61 to 0.97, with a median of 0.82 (IQR 0.76 to 0.86). The median C index was 0.82 for suicide models (IQR 0.74 to 0.85), 0.85 for non-fatal self-harm models (IQR 0.78 to 0.89), and 0.79 for composite outcome models (IQR 0.76 to 0.85).

Calibration was assessed for only 15 models (9%), using a calibration plot (*n* = 13) [5, 43–48] or calibration table (i.e. a table comparing predicted and observed outcome probabilities in strata of predicted risk, *n* = 2) [49, 50]. Calibration performance was mixed for these models and was sometimes assessed incorrectly (see Additional file 2: Supplementary results for details). None of the presented calibration plots were supplemented with a smoothed calibration curve or estimates of the calibration slope and intercept.

Clinical utility was assessed for seven models (one suicide death model reported in McCoy [51] and six self-harm models reported in Iorfino [52]). Both studies performed a decision curve analysis to evaluate the net benefit of the developed models compared to default strategies of treating everyone and treating no one. In both studies, the decision curve was used to guide the choice of threshold probabilities for decision-making, rather than determining a clinically reasonable range of thresholds a priori based on the relative harms of false positive and false negative classifications.

#### External validation

**Fig. 2:**
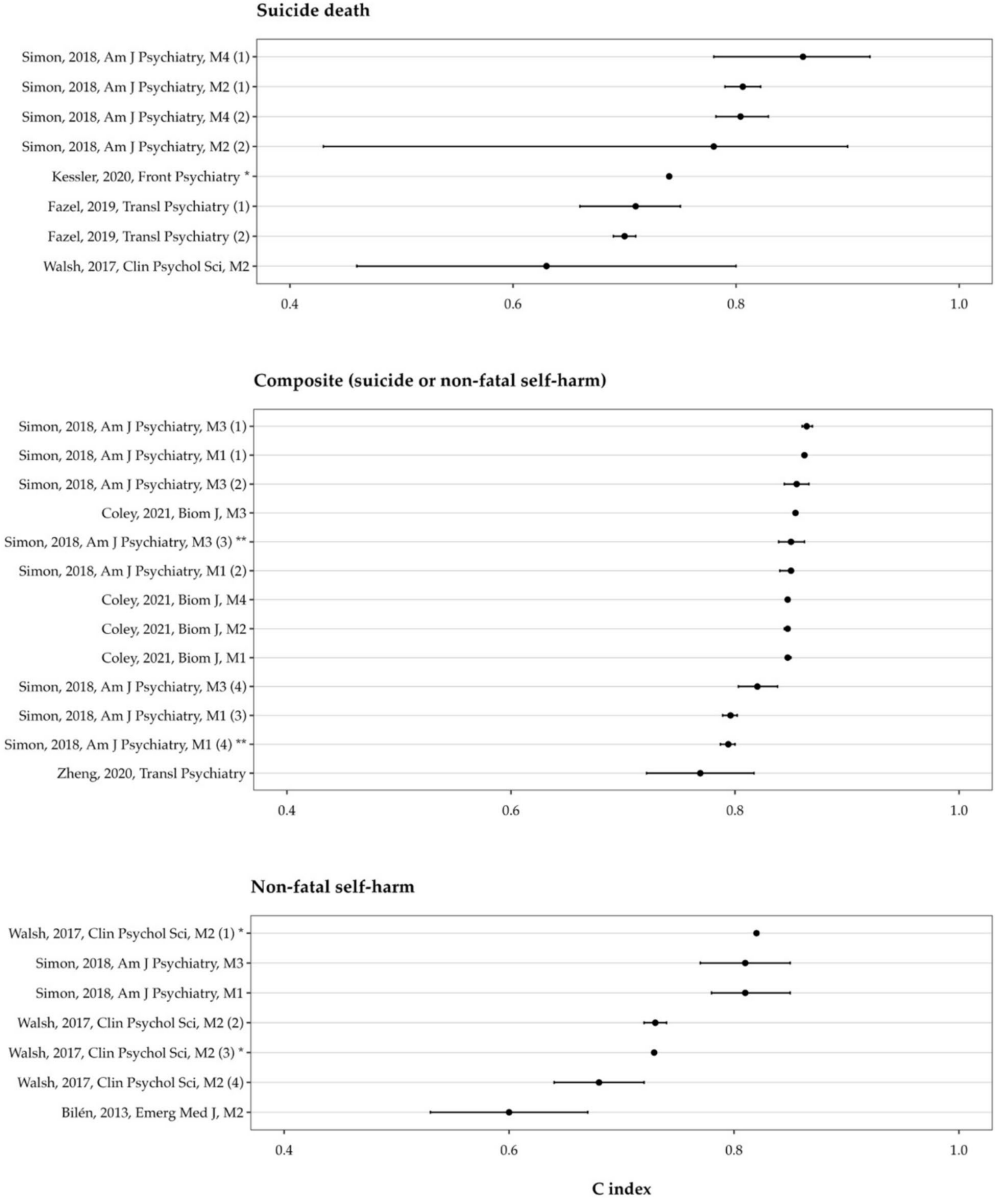
C indexes and 95% confidence intervals from external validations. Each row represents an external validation of the corresponding model on the *Y*-axis (see Additional file 4 for descriptions of the models). Error bars around some point estimates are not visible due to the confidence interval being too narrow (e.g. Coley, 2021, Biom J, M3). Numbers in parentheses indicate multiple external validations of the same model for the same outcome. Some models (e.g. Simon, 2018, Am J Psychiatry, M1; Walsh, 2017, Clin Psychol Sci, M2) were validated for predicting multiple outcomes. * Confidence intervals not reported. ** External validation with model updating

Almost all external validations (*n* = 28, 97%) reported a C index, which ranged from 0.60 to 0.86 (median 0.81, IQR 0.74 to 0.85). The median C index at external validation was 0.76 for models predicting suicide (IQR 0.71 to 0.80), 0.73 for models predicting non-fatal self-harm (IQR 0.70 to 0.81), and 0.85 for models predicting the composite outcome (IQR 0.82 to 0.85; see Fig. 2 and Additional file 4 for the C indexes and 95% CIs reported for externally validated models).

A measure of calibration was reported in nine external validations (31%; assessing six models). The OxMIS model—which predicts 1-year risk of suicide death in individuals with severe mental illness—was evaluated in two external validations [5, 53], both of which presented calibration plots. The model showed good overall calibration in both studies, with some overestimation of risk in patients with a higher observed probability of the outcome, although this overprediction was in a region with a sparse distribution of predicted probabilities (> 5% predicted risk, which only applied to 1.3% of the sample in the second external validation).

The models by Simon et al. [54] (predicting 90-day risk of suicide attempts and deaths following mental health specialty and general medical visits) were evaluated in an external validation study that presented calibration tables for all four models [55]. The two suicide attempt models showed good calibration, although they slightly overestimated risk for higher risk patients. The models for suicide death showed a similar pattern of miscalibration, with more extreme overestimation in the high predicted risk strata (e.g. two-to-four-fold overestimation of risk above the 99.5th percentile) [55]. The model for suicide attempts following general medical visits underpredicted risk in a subsequent external validation study conducted in a new centre [56], but this was improved following recalibration.

The Walsh model [57]—which was developed to predict 30-day risk of non-fatal suicide attempts—had two external validations that assessed calibration [56, 58]. The model showed extremely poor calibration in both studies, as indicated by the calibration slope and intercept [58] and the calibration plot [56]. However, calibration was improved in the second study [56] following recalibration using isotonic regression.

#### Discrimination performance stratified by model dimensionality and data source

Only models for which EPP could be calculated or approximated (*n* = 150) and external validations of these models (*n* = 15) were included in the post-hoc analysis stratified by model dimensionality. Of the 150 models, 48 (32%) were high-dimensional (EPP < 1), and the remaining 102 (68%) were low-dimensional (EPP ≥ 1). Only 2 of the 15 external validations (13%) evaluated the high-dimensional models. A C index was reported for 130 of the 150 models (87%) in the development study. The median C index was 0.82 (IQR 0.76 to 0.85) for high-dimensional models (*n* = 37) and 0.82 (IQR 0.75 to 0.86) for low-dimensional models (*n* = 93). Almost all of the external validations (14/15, 93%) reported a C index. The median C index at external validation was 0.76 (IQR 0.75 to 0.76) for high-dimensional models (*n* = 2) and 0.73 (IQR 0.70 to 0.85) for low-dimensional models (*n* = 12).

In the post-hoc analysis stratified by data source, the median C index at model development was 0.84 (IQR 0.76 to 0.86) for models based on routine data (*n* = 89) and 0.81 (IQR 0.75 to 0.86) for models based on prospectively collected data (*n* = 58). Nearly all external validations (*n* = 27) were conducted using routine data, with a median C index of 0.81 (IQR 0.76 to 0.85). Only one external validation was based on data from a prospective cohort study, which had a C index of 0.60.

### Risk of bias

**Fig. 3:**
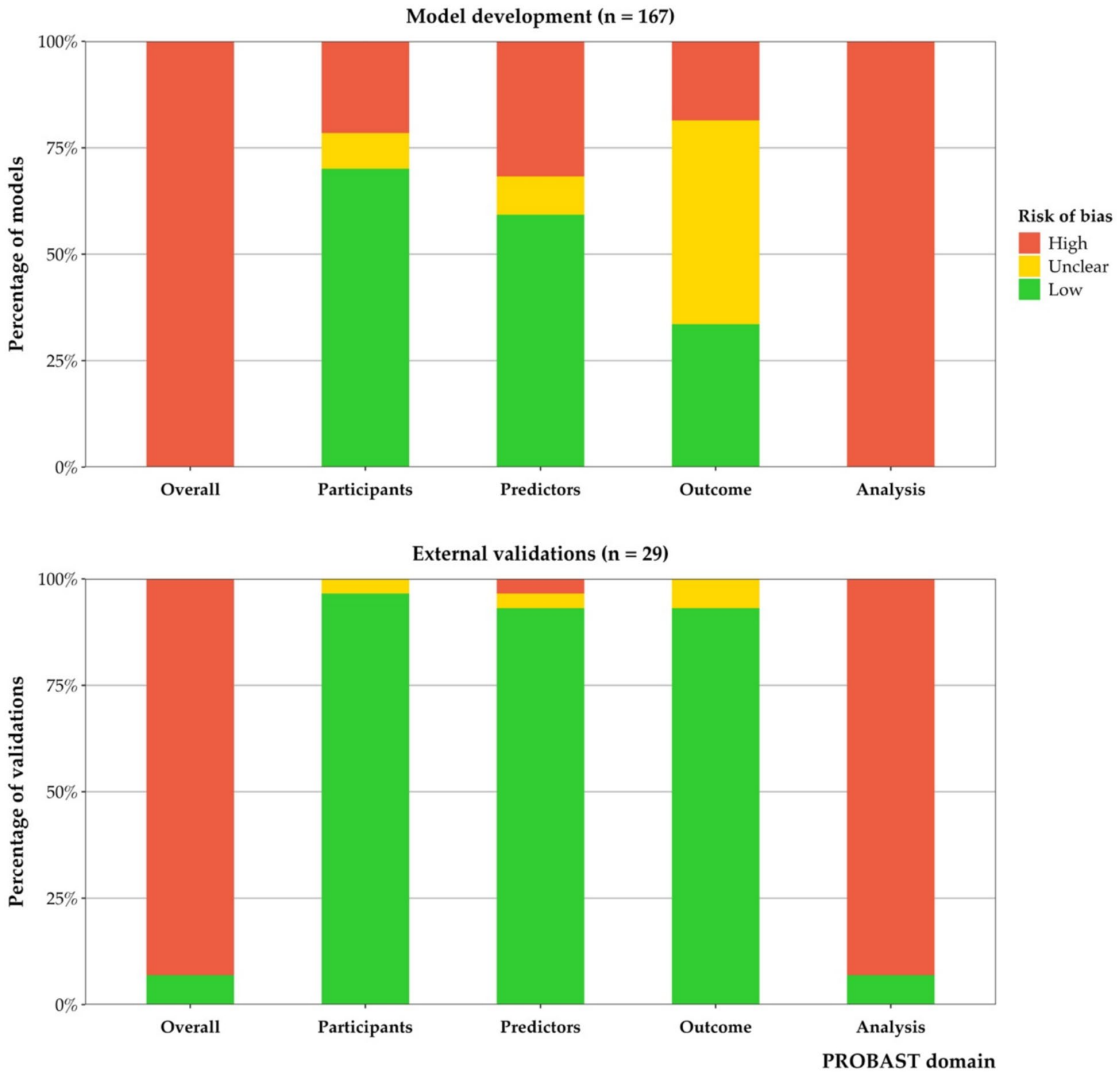
Risk of bias ratings according to PROBAST (overall and by domain) for all developed models (*n* = 167) and external validations (*n* = 29)

**Fig. 4:**
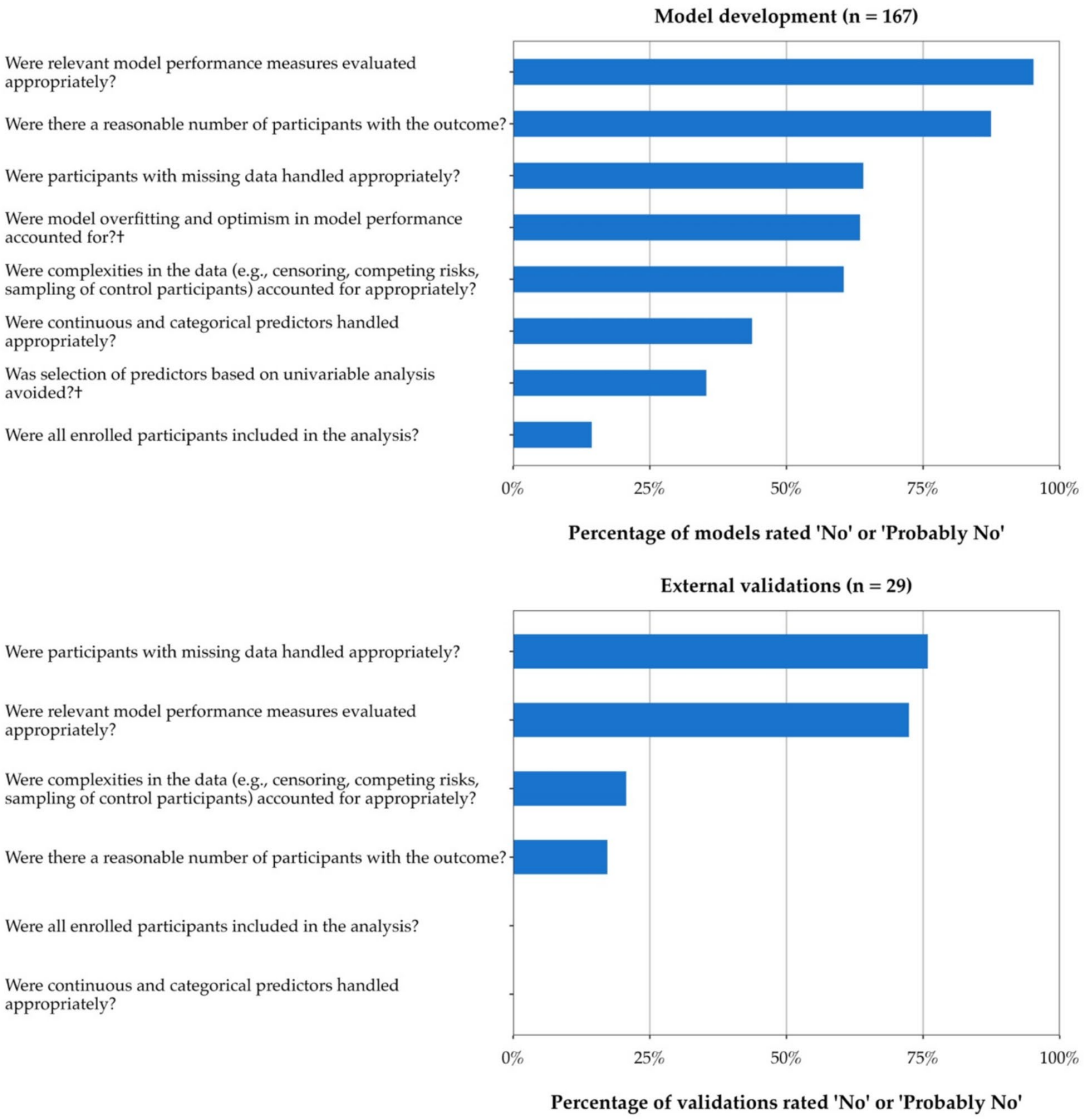
Common sources of bias in the PROBAST analysis domain for all developed models (*n* = 167) and external validations (*n* = 29). † Only applicable to model development studies

All developed models and almost all external validations (*n* = 27, 93%) were at high risk of bias according to PROBAST (Fig. 3). Only two external validations [5, 53], both evaluating the OxMIS model [5], were at low risk of bias. The overall PROBAST rating was mainly driven by the assessment of the analysis domain (see Fig. 4 and Table 4 for details). Common issues in the other PROBAST domains included inappropriate inclusion/exclusion criteria or recruitment strategy (making study participants unrepresentative of the model’s intended target population), unavailability of some predictors at the intended time of model use, outcome mis-ascertainment (assessed using a method with high levels of measurement error), outcomes not being determined in a similar way for all participants, and the time interval between predictor assessment and outcome determination being unclear or inappropriate (e.g. studies where predictor values were assessed up to the time of event). More details are provided in Additional file 2: Supplementary results and Additional file 5.

**Table 4:** Common methodological shortcomings of self-harm and suicide prediction models based on the PROBAST analysis domain

All developed models and 93% of external validations were rated at high risk of bias on the PROBAST analysis domain. There were only two external validations rated at low risk of bias for this domain. The most common issue was incomplete evaluation of model performance, which applied to almost all developed models and external validations (*n* = 180, 92%) because of no or incorrect assessment of calibration. This made it challenging to judge the accuracy of the models’ absolute predicted probabilities. Small sample size was another frequent problem, with 151 models and validations (77%) having an insufficient number of participants with the outcome according to PROBAST (defined as the EPP being < 10 for model development studies, and the number of participants with the outcome being < 100 for model validation studies [29], although these sample size recommendations have been updated in more recent methodological work [34, 72, 73, 110]). Insufficient sample sizes increase the risk of overfitting and optimism for model development studies [34], and can lead to imprecise estimates of model predictive performance for validation studies [110, 130]. Issues with reporting and handling of missing data were also common (*n* = 129, 66%). For instance, 63 models and validations (32%) made no explicit mention of methods to handle missing data, 28 (14%) used unclear methods, and 24 (12%) used complete-case analysis. Risk of bias due to missing data increases with increasing proportion of missingness [131]. However, most models and validations (*n* = 108, 55%) did not report any information about the amount of missing data. Only 40 models and validations (20%) clearly reported the proportion of missingness for all variables. Many development studies did not fully account for overfitting and optimism in the model performance estimates (*n* = 106, 63%). These studies either did not perform any internal validation, used inadequate methods (e.g. a single random split), or applied bootstrapping or cross-validation techniques without replicating all the model development steps (e.g. the variable selection). Over half of developed models and validations (*n* = 107, 55%) did not address one or more statistical complexities in the data that could affect the estimated model performance. Not accounting for censoring in the analysis was one of the most common issues, with several studies using a logistic regression approach when participants had different follow-up times (and the prediction horizon was unspecified), or excluding participants with incomplete follow-up from the analysis. Another common issue was using data from a nested case–control or case-cohort sample for model development without adjusting for the sampling fractions, and/or applying class imbalance correction methods during the analysis without recalibrating the model following development. The resulting probability estimates from such models will be distorted (often strongly overestimated) [41, 84, 85, 132]. Finally, some studies used data that could include multiple observations per person (e.g. hospitalisations or visits) for model development and/or validation. However, most did not account for clustering by using multi-level or random-effects modelling methods. Other shortcomings in the analysis domain included issues with the handling of continuous and categorical predictors (*n* = 73, 37%), selection of predictors based on univariable analysis prior to multivariable modelling (*n* = 59, 35%), and not including all enrolled participants in the analysis (*n* = 24, 12%). **Model development versus external validation studies** Overall, the quality of external validations was higher than model development studies (Figs. 3 and 4). Only 17% of developed models (*n* = 28) were rated at low risk of bias on all PROBAST domains except for analysis, compared with 79% of external validations (*n* = 23). In the analysis domain, handling of missing data was the most common issue for external validations (76% of validations vs 64% of developed models). However, all other signalling questions in this domain were less problematic for external validations than development studies. For instance, a higher proportion of external validations correctly evaluated both model calibration and discrimination compared to development studies (28% vs 5%), had adequate sample sizes (83% vs 13%), and correctly addressed statistical complexities in the data such as censoring and clustering of observations (79% vs 40%). Furthermore, all 29 external validations handled continuous and categorical predictors correctly (compared to 56% of developed models), and none inappropriately excluded participants from the analysis (compared to 14% of developed models).	

**Table:** 

**What is already known on this topic** • Several prognostic models are available to predict the risk of self-harm and suicide, but their usefulness for suicide risk assessment is uncertain and has been widely debated. • To evaluate the potential for these models to improve clinical risk assessment, there is a need for high-quality systematic review evidence on their predictive performance and methodological quality. • Previous systematic reviews have been restricted to models developed using machine learning, used inappropriate measures to evaluate performance, or not sufficiently evaluated the risk of bias and reporting of existing models. **What this study adds** • The OxMIS model [5] (developed to predict 1-year risk of suicide in individuals with severe mental illness) and the Simon models [54] (developed to predict 90-day risk of suicide attempt and death following mental health specialty and general medical visits) showed good discrimination and calibration performance in external validation. New validations in more recent or local data are necessary to further assess robustness and generalisability. • Our findings challenge some expert opinion in the field that dismisses suicide risk models on the basis of their poor performance, and highlight the need for research looking at the translation of these models and their role in informing clinical practice. • Avoidable sources of research waste include methodological shortcomings in study design, conduct, and statistical analysis, lack of external validation studies, and inadequate reporting that prevents independent validation and clinical implementation of models.	

In addition to the issues covered by PROBAST, we identified a number of other methodological problems in the included studies, including correction for class imbalance, not accounting for clustering in visit-level data, and issues which affect the usability of a prediction model in real-world clinical practice (e.g. problems with definition of the prediction moment and/or prediction horizon, target population, and timing of predictor measurements). These methodological issues, along with specific examples, are listed in Table 5.

**Table 5:** Additional methodological issues identified in the included studies

Methodological issue	Example(s)	Impact on bias/model applicability
Model developed in data corrected for class imbalance	Sanderson et al. [46] developed a prediction model for suicide, using class weights to assign equal importance to both outcome classes (weights of 124/125 for individuals that died by suicide and 1/125 for those that did not). García de la Garza et al. [49] developed a suicide attempt prediction model using a form of under-sampling—balanced random forest—in which each classification tree is fitted to a bootstrap sample of the minority class and a random sample of the same size from the majority class. The impact of class imbalance correction on calibration can be seen in the calibration curves in Sanderson [46] (Figs. 1 and 2) and in the calibration table in García de la Garza et al. [49] (Table 1).	The model will show strong miscalibration (over-estimation of outcome risk) without better discrimination performance (see recent methodological work for a variety of modelling approaches [41, 84, 85]). Miscalibration reduces the clinical utility of the model [103] and can lead to incorrect treatment decisions, potentially causing patient harm [67].
Moment of prediction and/or prediction horizon defined retrospectively	Su et al. [111] used the time of first suicide attempt (for individuals with the outcome) and the last recorded clinical visit (for individuals without the outcome) as an index date, and defined several prediction windows of varying durations prior to this index date (with the model for a prediction window of X days only using data available at least X days prior to the index date). *	It is impossible to apply the model in clinical practice, as the intended moment of model use and prediction horizon cannot be defined in this way when using a model prospectively. The model can only be applied in retrospective data where the outcome status and its timing are known for all individuals.
Model developed in a target population which cannot be identified prospectively in real-world clinical practice (caused by inappropriate selection of cases and controls from the wider cohort in nested case–control datasets)	Walsh et al. [57, 112] and Lenert [113] defined cases as individuals with hospital episodes for non-fatal suicide attempts, and controls as a random sample of individuals with hospital episodes for other causes. The target population is all individuals who will have a hospital episode in the future, and the model is aiming to distinguish those who will have hospital episodes due to suicide attempts from those whose hospital episodes will be due to other causes. ^†^	The model is unusable in clinical practice, as it is unclear what target population it should be applied to (e.g. in the example, identifying individuals who will have hospital episodes in the future is not possible at the moment of prediction).
Model developed using predictor data collected up to the event time for individuals with the outcome and an arbitrary/matched time for individuals without the outcome	Barak-Corren et al. [114, 115] developed models using data collected up to but not including the first suicidal event for individuals with the outcome (and from all available time points for those without the outcome). **	The model is unusable in real-world settings, as it includes predictor information which would not be available at the time when the model would be used.
Excluding suicide deaths from the analysis when developing a model to predict self-harm or suicide attempt	Walsh et al. [57, 112] developed models for predicting non-fatal suicide attempts. They excluded all individuals who had died by suicide from the analysis. ^‡^ Many models also stated that they predicted the outcome of non-fatal self-harm or suicide attempts without clarifying whether there were any suicide deaths in the sample and how these were handled.	The model predictions relate to an unrealistic population where suicide deaths can never occur. Due to the extremely low prevalence of suicide, the absolute value of predictions will not be significantly different to a composite outcome model. However, the interpretation of predictions is problematic—the model is predicting the probability of having a non-fatal self-harm episode over a specific period, assuming that the individual does not die of suicide during that time. When developing a prediction model for self-harm, unless there are no suicide deaths in the prediction horizon of interest, it is more clinically meaningful to predict a composite outcome (fatal or non-fatal self-harm).
Not accounting for clustering during model development (applicable when each individual may contribute multiple visits and events to the analysis)	Coley et al. [116] developed prediction models for suicide death within 90 days of outpatient mental health visits. The analysis could include multiple visits per person, but this was not accounted for by using multi-level or random-effects modelling methods. There is also potential for overlap in prediction windows and double-counting of events for visits close in time (e.g. the same suicide death falling within 90 days of two separate visits). Another study by Coley et al. [117] specifically evaluated the impact of clustering on the performance of a prediction model for suicide attempts following outpatient mental health visits (see Additional file 2: Supplementary results).	Not correctly accounting for clustering can lead to underestimation and bias in predictor effects [29]. To our knowledge, the extent to which double-counting events in this context may introduce bias in predictive performance estimates has not been evaluated before. We are also not aware of any studies addressing sample size requirements for developing and validating prediction models in clustered data. More methodological work is required to establish good statistical practice in this area.

^*^ See [47, 57, 77, 111–115, 118–127] for further example

^†^ See [57, 112, 113, 121, 128, 129] for further examples

^**^ See [111, 114, 115, 120, 121, 125–127] for further examples

^‡^ See [57, 58, 112, 113, 124] for further examples

## Discussion

In this systematic review of 167 models and 29 external validations, we have provided a comprehensive overview and field synopsis of prognostic risk models for suicide and self-harm, summarised their predictive performance, and critically appraised their methodological quality and risk of bias. The vast majority of studies focused on model development. External validations were uncommon, with less than 10% of all developed models having been externally validated, although more have been published over the past 5 years. Over 80% of developed models were not publicly available in a format that would enable independent validation or use by others. Many of the identified models showed good discriminative ability, and the range of reported C indexes (with a median of 0.81 in external validation) was comparable to models in other areas of medicine (cardiovascular, respiratory, COVID-19) [30, 31, 59], suggesting that pessimism about the feasibility of suicide risk prediction is not evidence-based. However, calibration was only assessed in a minority of studies. We identified five models, three for predicting suicide deaths [5, 54] and two for predicting the composite outcome of suicide attempt or death [54] with adequate discrimination and calibration performance in external validation. Overall, discrimination performance was comparable between models developed using routine and prospectively collected data, and between high-dimensional (EPP < 1) and low-dimensional (EPP ≥ 1) models.

Incomplete reporting was common, and the PROBAST assessment suggested that there were some methodological concerns in almost all model development and external validation studies, particularly incomplete assessment of model performance (ignoring calibration), insufficient sample sizes, issues with reporting and handling of missing data, and not adequately accounting for overfitting and optimism in predictive performance estimates. Despite this, among the very few externally validated models, some did not show clear evidence of optimism when comparing the estimated C indexes from model development and external validation studies (Additional file 4). Overall, the methodological quality of external validations was higher than model development studies.

### Comparison with previous reviews

Several systematic reviews of self-harm and suicide prediction models have been published in recent years [13–15, 60–66]. However, the most recent reviews have been restricted to models developed using machine learning methods [14, 15, 60, 61, 63–66]. Furthermore, some reviews have focused on inappropriate statistical measures to evaluate model predictive performance. For instance, two recent reviews [14, 62] have presented odds ratios in their meta-analyses, which is a measure of association rather than predictive performance. Other reviews [13, 15, 60, 61, 63–66] have focused entirely on measures of classification and discrimination without mentioning model calibration, despite the importance of well-calibrated risk predictions for clinical decision making [67]. Finally, some of these reviews have not performed any risk of bias assessment [61, 66], and many have used inappropriate tools not designed for prediction modelling studies [13, 14, 60, 62, 63, 65]. In the two reviews using PROBAST [15, 64], a higher proportion of studies (22–25%) were rated at low risk of bias compared to our review. However, one of these reviews [15] used an adaptation of PROBAST created by the authors, which included a reduced number of items and modifications in some domains. In the other review [64], the authors did not present any information on the risk of bias ratings for each domain and their rationale.

### Reporting and methodological issues

Most included studies did not fully conform to current reporting guidelines. Information on study characteristics, methodological details, and predictive performance measures was missing from many papers, and 83% of developed models were not made available in a format to allow predictions in new individuals, not making it possible for others to evaluate or implement them. Only 11 of the 91 included studies (12%) mentioned adherence to the TRIPOD reporting guidelines [68–70], despite most (79%) being published after TRIPOD [68, 69] first became available in 2015. Incomplete and poor reporting makes it challenging to synthesise the performance of models and appraise their risk of bias; it also prevents future studies from building on previous work, contributing to large amounts of research waste [31, 71].

The most common methodological concern according to PROBAST was not assessing model calibration. Only a minority of studies evaluated calibration, and a few reported that calibration was assessed without presenting any results from the assessment. Inadequate sample size was another concern, particularly in model development studies. Sample size for model development must be large enough to minimise risk of overfitting and ensure precise predictions [34]. Guidelines for calculating the minimum required sample size for prediction model development [34] and external validation [72, 73] are available. Inappropriate or unclear handling of missing data also occurred frequently, with many studies not reporting how missing data were handled, and some using complete-case analysis, which can lead to reduced precision and biased model performance estimates [29].

Internal validation was done sub-optimally in many studies. The most commonly used approach was randomly splitting the sample into training and testing sets, which is inefficient and does not adequately adjust for optimism in model performance [74, 75]. Studies that used bootstrapping or cross-validation did not always replicate the exact model-building strategy. For instance, some machine learning-based investigations used cross-validation techniques for hyperparameter tuning during model development but failed to replicate this during internal validation (e.g. by using a nested cross-validation procedure) [42, 46, 47, 76–80]. Such approaches can underestimate the amount of optimism in model performance [29, 81].

Statistical complexities in the data were not appropriately handled in several studies. For instance, some used a logistic regression approach for model development when participants had different follow-up times, and the prediction horizon was not clearly defined. This approach is problematic as the predictions do not have a specific time period, and the duration of follow-up is ignored [29]. For predicting long-term prognostic outcomes where the follow-up time for some individuals is censored, a time-to-event analysis should be used to correctly account for censoring. Another common issue was developing a model in nested case–control or case-cohort data without appropriately adjusting for the sampling fractions from the original cohort (i.e. reweighting the cases and controls by the inverse of their sampling fraction), which leads to over-estimated predicted probabilities [29, 82, 83]. This problem also occurred in studies that corrected the dataset for class imbalance prior to model development (e.g. using random under- or over-sampling). As has been shown in recent methodological work for a variety of modelling approaches [41, 84, 85], developing models on imbalance-corrected data leads to strong miscalibration (over-estimation of the outcome risk) without resulting in better discrimination performance.

Finally, we identified a number of issues in the design of some model development studies, which affected the usability of the model in real-world clinical practice (Table 5). These included defining the moment of prediction and/or prediction horizon retrospectively (using the event time as reference), using data recorded up to the event time to make predictions, or defining a target population that cannot be identified prospectively in a clinical setting (e.g. nested case–control studies that used inappropriate criteria for selection of cases and controls from the wider cohort). Such models can only be applied in retrospective datasets, making them clinically unusable.

### Implications for practice and recommendations for future research

Our systematic review has identified a number of models for prediction of self-harm and suicide risk with good discrimination and calibration performance in external validation: the OxMIS model [5] (developed to predict 1-year risk of suicide in individuals with severe mental illness), and the Simon models [54] (developed to predict 90-day risk of suicide attempt and death following mental health specialty and general medical visits). The OxMIS model had two external validations [5, 53] rated at low risk of bias according to PROBAST. We recommend that future studies further validate these models in more recent or local data to assess their robustness and generalisability. Evidence from our systematic review can also be considered in future updates of clinical guidelines, including the NICE self-harm guidance [11] and the suicide prevention guidance by NHS England [12], potentially informing their recommendations regarding the use of suicide prediction models [7].

Despite the publication of so many studies in this area, very few models are actually used in clinical practice, suggesting a major gap between the development and implementation of suicide prediction models. Although models should not be prematurely implemented without adequate validation, many are published with no intention of being used clinically. Notably, only 7% of developed models were presented in a format that was aimed at the end user (i.e. a prediction tool such as an online calculator or point score system). We also identified some models that were impossible to apply prospectively, rendering them clinically unusable. Presentation of model outputs in a user-friendly format and the involvement of users (e.g. clinicians and patients) can help address this implementation gap and improve collaborative approaches to risk assessment. To support the implementation of models that show good predictive performance and evidence of clinical usefulness, future research should also focus on assessing the cost-effectiveness of promising models and their impact on clinical decision making and patient outcomes [86, 87]. The wider context of improving clinical care and introducing a culture of safety is an important component to examining these outcomes in addition to improving assessment [7].

Only a small proportion of the identified models (less than 10%) were externally validated. The development of so many suicide prediction models, often using sub-optimal methods, and many answering the same research question, is a significant source of research waste. In our view, the focus of the field should shift more towards external validation of existing models and comparing their predictive performance, ideally using head-to-head comparisons of competing models in the same dataset. Our review identified only one such study [56]. With more external validation work, it will be possible for future research to meta-analyse the predictive performance of competing models and identify sources of heterogeneity in their performance across different settings and populations [32, 88, 89].

Our review includes prediction models developed using both routine and prospectively collected research data; models cannot be assumed to generalise across different data sources and settings, even if the population of interest is the same [86, 90]. For instance, a model developed using predictors based on administrative or insurance claims data may not be generalisable to settings where predictors are systematically measured using standardised assessments. Potential challenges to model transportability across different data sources and settings include differences in the extent of measurement error in coding predictors [91] and potential biases inherent in routine data (e.g. low sensitivity of diagnostic codes, missingness patterns systematically related to illness severity and associated health service use) [92–94]. Therefore, generalising predictive performance estimates across different data sources requires caution, and a new targeted validation may be necessary to assess model transportability using data representative of the setting of intended use [90].

When a model shows poor predictive performance in new patients, researchers should consider refining and updating the existing model with the data from new individuals where possible, before developing a new model from scratch [95]. Updating models is a more efficient use of the available evidence base and can provide more robust and stable predictions, since it generally involves estimation of fewer parameters compared to developing a new model [96]. There are several approaches that differ in the extensiveness of updating, from simple recalibration methods (e.g. adjusting the model intercept, with or without recalibration of the regression coefficients using a single adjustment factor) to more extensive revision methods (e.g. adjustment of specific predictors effects, re-estimating the regression coefficients of all predictors, or even addition of new predictors) [95]. In general, when the model shows good discrimination but poor calibration in new patients (e.g. systematic over- or under-estimation of risk), simple recalibration approaches can often improve the calibration to a similar extent as more complex revision methods that estimate more parameters. However, if the model’s discriminative ability needs to be improved, then more extensive updating with revision methods may be necessary [95]. To enable external validation and updating by independent investigators, complete, accurate, and transparent reporting of the original development studies is key [70]. Therefore, future studies should adhere to the TRIPOD + AI reporting guidelines [70] (https://www.tripod-statement.org/), and always provide their final developed model in a format that can be used by others to make individualised predictions.

In some circumstances, the development of a new prediction model may be warranted (for example, because there are no suitable models for the target population or setting of interest, or due to unavailability of the predictors from a promising model in a particular setting). However, this should always be motivated by clinical need (i.e. a clearly defined target population and setting, intended moment of model use, and prediction horizon), rather than the availability of datasets or analytic methods. Before developing a new model, researchers should ensure that their target population can be identified in real-world clinical practice and the model can be used prospectively (rather than only being applicable to retrospective data where the outcome status and its timing is known for all individuals). Models should be developed in sufficiently large samples [34] and using appropriate methodology [29, 97, 98]. Nearly all the potential sources of bias that we identified in the literature were avoidable issues in study design, conduct, and statistical analysis. These can be addressed by improved methodological rigour and following best-practice guidance, as highlighted in this paper.

Finally, when reporting model performance, researchers should focus not only on measures of discrimination and classification, but also on calibration performance (preferably using flexible calibration curves) [67] and clinical utility (for example, by calculating the net benefit of the model) [99–102]. Poorly calibrated models can provide misleading information to clinicians and patients, and may lead to over- or under-treatment, limiting the clinical usefulness of the model [67, 103]. Despite the importance of these performance criteria for assessing models intended to support clinical decision making [104], they are largely neglected in the suicide prediction literature. To ensure that a developed model’s predictions are robust and reliable in new individuals, researchers should also consider examining the stability of their models (i.e. the extent to which predicted risks for a given individual may differ depending on the particular sample used for model development). Methods for quantifying the (in)stability of prediction models at the development stage have recently been proposed [33].

### Strengths and limitations

This comprehensive systematic review of prediction models for self-harm and suicide includes models developed using both machine learning and regression-based methods, and has been conducted following the most recent methodological guidance, including for data extraction and evaluation of risk of bias. However, our study has a number of limitations. First, our search covers studies published up to December 2021, with an update in October 2024 to include new external validations of the included models. The number of published prediction models for self-harm and suicide has grown substantially in recent years. A rigorous review and critical appraisal of these studies is time-consuming and requires periodic updating. Given the large number of models and validations included in our review, it is unlikely that the addition of newer studies would alter our main conclusions and recommendations for future research. Nevertheless, there may be one or more newly developed models with good predictive performance and low risk of bias that have been published since our original search date.

Second, suicide risk assessment scales, checklists, and tools that were not empirically derived or not based on statistically derived weights were outside the scope of this review. Although some risk assessment scales are used in clinical practice [105], we decided not to include these as many methodological issues related to model development, validation, and predictive performance (e.g. calibration) do not apply. Other work has demonstrated mixed discrimination for such risk scales [106, 107].

Third, most of the screening, data extraction, and risk of bias assessment was completed by a single reviewer, with 10% of references/included studies independently assessed by a second reviewer at each stage. In particular, the assessment of several PROBAST signalling questions is somewhat subjective and may have been affected by lack of duplicate assessment. The rationale for all risk of bias ratings has been provided in Additional file 5.

Finally, PROBAST is primarily aimed at assessing the risk of bias for regression-based models. Some specific issues related to machine learning modelling techniques (e.g. due to differences in variable selection approaches, adjustment for overfitting, etc.) [29, 108] are not covered in the current version but are addressed in an adaptation of the tool currently under development (PROBAST-AI) [108]. Furthermore, since the publication of PROBAST in 2019, there have been multiple methodological advances in the field, including recommendations for handling missing data [109] and sample size calculations for model development [34] and external validation [72, 73, 110], which should be considered in future updates.

## Conclusions

Although several prognostic risk models for self-harm and suicide have been developed and many show good discriminative ability, these often answer the same research question, and over 90% have not been externally validated, leading to considerable research waste. Other avoidable sources of waste include methodological shortcomings and inadequate reporting that prevents models from being independently validated and implemented in clinical practice. To address these sources of waste, future research should focus more on external validation and head-to-head comparisons of competing models, updating and recalibration of existing models, and extending them with new predictors. New model development work should only be considered if addressing a clear clinical need for which no suitable models are available. Studies should follow methodological guidance for the development and evaluation of prediction models and should be reported fully, accurately, and transparently. Our review has identified five models for predicting risk of self-harm and suicide with good predictive performance in external validation. These should be further validated in local settings, followed by recalibration and updating if necessary. In summary, our review suggests that blanket criticisms of the predictive performance of risk models for suicide outcomes are not evidence-based. 

## Supplementary Information

Additional file 1. Completed TRIPOD-SRMA Checklist for reporting systematic reviews of prediction model studies.

Additional file 2. Supplementary methods and results, Tables S1–S2, and Figs. S1–S4. Table S1 Countries from which data were used for model development and external validation. Table S2 Additional characteristics of included models and external validations. Fig. S1 Histogram of the number of events per candidate predictor parameter (EPP) based on the 150 models for which EPP could be calculated or approximated. Fig. S2 C indexes and 95% confidence intervals of suicide death models from development studies. Fig. S3 C indexes and 95% confidence intervals of composite outcome models from development studies. Fig. S4 C indexes and 95% confidence intervals of non-fatal self-harm models from development studies.

Additional file 3. List of all reviewed models and validations, with summary information on the target population, outcome, predictive performance, risk of bias, and model presentation.

Additional file 4. Discrimination performance results and reported classification metrics for all reviewed models, presented separately by validation type (apparent performance, internal validation, external validation).

Additional file 5. PROBAST ratings and rationale (overall and by domain) for all reviewed models and validations.

Additional file 6: R script for statistical analyses reported in the manuscript.

## Data Availability

The datasets used during the current study are available from the corresponding author on reasonable request. R code is provided in Additional file 6.

## Abbreviations

PROBAST: Prediction model Risk Of Bias ASsessment Tool
TRIPOD-SRMA: Transparent reporting of multivariable prediction models for individual prognosis or diagnosis checklist for systematic reviews and meta-analyses
CHARMS: CHecklist for critical Appraisal and data extraction for systematic Reviews of Prediction Modelling Studies
CI: Confidence interval
IQR: Interquartile range
ICD: International Classification of Disease
EPP: Events per candidate predictor parameter
LASSO: Least absolute shrinkage and selection operator
SMOTE: Synthetic Minority Oversampling Technique
PRISMA: Preferred Reporting Items for Systematic Reviews and Meta-analyses
