# Supplementary material for: Prediction models for self-harm and suicide: a systematic review and critical appraisal
Source: BMC Med. 2025 Oct 9;23:549. doi: 10.1186/s12916-025-04367-6 (PMC12513157; doi:10.1186/s12916-025-04367-6)
Supplement: Supplementary file 2 — Additional file 2. Supplementary methods and results, Tables S1–S2, and Figs. S1–S4. Table S1 Countries from which data were used for model development and external validation. Table S2 Additional characteristics of included models and external validations. Fig. S1 Histogram of the number of events per candidate predictor parameter (EPP) based on the 150 models for which EPP could be calculated or approximated. Fig. S2 C indexes and 95% confidence intervals of suicide death models from development studies. Fig. S3 C indexes and 95% confidence intervals of composite outcome models from development studies. Fig. S4 C indexes and 95% confidence intervals of non-fatal self-harm models from development studies. [file 12916_2025_4367_MOESM2_ESM.docx]

# Supplementary methods

## Deviations from PROSPERO protocol

We planned to extract information on the ratio of the total number of observed (O) to expected (E) events as a measure of calibration. The total O:E ratio is a crude measure of overall model calibration across the entire range of predicted probabilities, and is commonly used in meta-analyses of prediction model studies when other measures of calibration are not presented [88]. In our review, the studies for which this statistic was derivable also presented a calibration plot or table. Since we did not perform a meta-analysis, we decided to provide a narrative summary of calibration performance based on the measures reported in the original studies, rather than the total O:E ratio.

In addition, we planned to report classification measures (including sensitivity, specificity, positive and negative predictive values) where reported. However, the probability thresholds used to calculate these statistics varied considerably across included studies. Since the values of classification measures are strongly dependent on the chosen probability thresholds [17], we decided that comparing these measures across different studies and models would not be meaningful. The list of classification measures reported for each model—including whether they relate to apparent, internal, or external validation performance—is provided in Additional file 4.

Our protocol stated that we would consider conducting a meta-analysis, followed by meta-regression analyses to investigate sources of heterogeneity in model performance. However, given the extent of heterogeneity in the identified studies (in relation to predicted outcomes, prediction horizons, predictors, target populations and settings), and the broad scope of our review (identifying all prognostic models for suicide death and/or non-fatal self-harm), we considered meta-analysis not to be of added value. Furthermore, recent methodological guidance on systematic reviews of prognostic model studies [32], which became available after our protocol registration on PROSPERO, recommends meta-analysis only if there are more than five external validation studies for the same index model. However, our review did not identify any models with more than five validation studies.

## Search strings

**MEDLINE**

**Search terms for suicide:**

1 exp Suicide/ OR Suicid*.ti,ab,kw.

**Search terms for self-harm:**

2 (self$harm* OR self-harm*).ti,ab,kw.

3 (exp Self-Injurious Behavior/ OR self$injur*.ti,ab,kw. OR self-injur*.ti,ab,kw.)

4 (self$poison* OR self-poison*).ti,ab,kw.

5 (para$suicid* OR para-suicid*).ti,ab,kw.

6 (exp Self Mutilation/ OR self$mutilat*.ti,ab,kw.)

7 (exp Drug Overdose/ OR overdos*.ti,ab,kw.)

8 ((auto ADJ (aggress* OR mutilat*)) OR (autoaggress* OR automutilat*)).ti,ab,kw.

9 ((self OR themsel* OR oneself*) ADJ2 (harm* OR cut* OR immolat* OR inflict* OR injur* OR mutilat* OR poison* OR damag* OR destruct*)).ti,ab,kw.

10 2 OR 3 OR 4 OR 5 OR 6 OR 7 OR 8 OR 9

**Search terms for predictive performance measures:**

11 (exp "Sensitivity and Specificity"/ OR sensitivit*.ti,ab,kw. OR specificit*.ti,ab,kw.)

12 (exp "Predictive Value of Tests"/ OR (predict* ADJ3 value*).ti,ab,kw. OR PPV.ti,ab,kw. OR NPV.ti,ab,kw.)

13 ((pre$test OR pre-test OR post$test OR post-test) ADJ2 probabilit*).ti,ab,kw.

14 ((pre$test OR pre-test OR post$test OR post-test) ADJ2 odd*).ti,ab,kw.

15 (likelihood ADJ3 ratio*).ti,ab,kw.

16 (Diagnostic odds ratio).ti,ab,kw.

17 ((c$statistic* OR C-statistic*).ti,ab,kw. OR (c$index* OR C-index*).ti,ab,kw. OR (d$statisic* OR D-statistic*).ti,ab,kw. OR (exp Area Under Curve/ OR exp ROC Curve/ OR (area under ADJ2 curve).ti,ab,kw. OR AUC.ti,ab,kw. OR receiver operating characteristic.ti,ab,kw. OR (roc ADJ2 (analy* OR curv* OR plot*)).ti,ab,kw.))

18 (Calibrat* OR Brier score OR (Hosmer ADJ1 Lemeshow)).ti,ab,kw.

19 (Net reclassification improvement OR net reclassification index OR NRI).ti,ab,kw.

20 (Integrated discrimination improvement OR IDI).ti,ab,kw.

21 11 OR 12 OR 13 OR 14 OR 15 OR 16 OR 17 OR 18 OR 19 OR 20

**Search terms for risk prediction models:**

22 (exp Risk Assessment/ OR risk assessment.ti,ab,kw.)

23 exp Decision Support Techniques/

24 exp Checklist/

25 (Predict*.ti. OR Rule*.ti,ab,kw.)

26 (Predict* AND Model*).ti,ab,kw.

27 (Decision*.ti,ab,kw. AND (Model*.ti,ab,kw. OR Clinical*.ti,ab,kw. OR exp Logistic Models/))

28 (Prognostic AND (Scor* OR Model*)).ti,ab,kw.

29 ((screen* OR assess* OR predict* OR prognos*).ti,ab,kw. AND (exp Algorithms/ OR algorithm*.ti,ab,kw. OR tool*.ti,ab,kw. OR scale*.ti,ab,kw. OR rule*.ti,ab,kw. OR checklist*.ti,ab,kw. OR rating*.ti,ab,kw. OR score*.ti,ab,kw. OR index.ti,ab,kw. OR indices.ti,ab,kw. OR model*.ti,ab,kw. OR machine learning.ti,ab,kw. OR decision tree.ti,ab,kw.))

30 (risk.ti,ab,kw. AND (exp Algorithms/ OR algorithm*.ti,ab,kw. OR tool*.ti,ab,kw. OR scale*.ti,ab,kw. OR rule*.ti,ab,kw. OR checklist*.ti,ab,kw. OR rating*.ti,ab,kw. OR score*.ti,ab,kw. OR index.ti,ab,kw. OR indices.ti,ab,kw. OR model*.ti,ab,kw. OR machine learning.ti,ab,kw. OR exp Decision Trees/ OR decision tree.ti,ab,kw.))

31 22 OR 23 OR 24 OR 25 OR 26 OR 27 OR 28 OR 29 OR 30

32 (1 OR 10) AND 21 AND 31

**EMBASE**

**Search terms for suicide:**
1 exp suicide/ OR Suicid*.ti,ab,hw.

**Search terms for self-harm:**

2 exp self immolation/

3 (self$harm* OR self-harm*).ti,ab,hw.

4 (self$injur* OR self-injur*).ti,ab,hw.

5 (exp self poisoning/ OR self$poison*.ti,ab,hw. OR self-poison*.ti,ab,hw.)

6 (exp suicide attempt/ OR para$suicid*.ti,ab,hw. OR para-suicid*.ti,ab,hw.)

7 (exp automutilation/ OR self$mutilat*.ti,ab,hw.)

8 (exp drug overdose/ OR overdos*.ti,ab,hw.)

9 ((auto ADJ (aggress* OR mutilat*)) OR (autoaggress* OR automutilat*)).ti,ab,hw.

10 ((self OR themsel* OR oneself*) ADJ2 (harm* OR cut* OR immolat* OR inflict* OR injur* OR mutilat* OR poison* OR damag* OR destruct*)).ti,ab,hw.

11 2 OR 3 OR 4 OR 5 OR 6 OR 7 OR 8 OR 9 OR 10

**Search terms for predictive performance measures:**

12 ("sensitivity and specificity"/ OR sensitivit*.ti,ab,hw. OR specificit*.ti,ab,hw.)

13 (exp predictive value/ OR (predict* ADJ3 value*).ti,ab,hw. OR PPV.ti,ab,hw. OR NPV.ti,ab,hw.)

14 ((pre$test OR pre-test OR post$test OR post-test) ADJ2 probabilit*).ti,ab,hw.

15 ((pre$test OR pre-test OR post$test OR post-test) ADJ2 odd*).ti,ab,hw.

16 (likelihood ADJ3 ratio*).ti,ab,hw.

17 (Diagnostic odds ratio).ti,ab,hw.

18 ((c$statistic* OR C-statistic*).ti,ab,hw. OR (c$index* OR C-index*).ti,ab,hw. OR (d$statisic* OR D-statistic*).ti,ab,hw. OR (exp area under the curve/ OR exp receiver operating characteristic/ OR (area under ADJ2 curve).ti,ab,hw. OR AUC.ti,ab,hw. OR receiver operating characteristic.ti,ab,hw. OR (roc ADJ2 (analy* OR curv* OR plot*)).ti,ab,hw.))

19 (Calibrat* OR Brier score OR (Hosmer ADJ1 Lemeshow)).ti,ab,hw.

20 (Net reclassification improvement OR net reclassification index OR NRI).ti,ab,hw.

21 (Integrated discrimination improvement OR IDI).ti,ab,hw.

22 12 OR 13 OR 14 OR 15 OR 16 OR 17 OR 18 OR 19 OR 20 OR 21

**Search terms for risk prediction models:**

23 (exp risk assessment/ OR risk assessment.ti,ab,hw.)

24 exp decision support system/

25 exp checklist/

26 (Predict*.ti. OR Rule*.ti,ab,hw.)

27 (Predict* AND Model).ti,ab,hw.

28 (Decision* AND (Model* OR Clinical*)).ti,ab,hw.

29 (Prognostic AND (Scor* OR Model*)).ti,ab,hw.

30 ((screen* OR assess* OR predict* OR prognos*).ti,ab,hw. AND (exp algorithm/ OR algorithm*.ti,ab,hw. OR tool*.ti,ab,hw. OR scale*.ti,ab,hw. OR rule*.ti,ab,hw. OR checklist*.ti,ab,hw. OR rating*.ti,ab,hw. OR score*.ti,ab,hw. OR index.ti,ab,hw. OR indices.ti,ab,hw. OR model*.ti,ab,hw. OR exp machine learning/ OR machine learning.ti,ab,hw. OR decision tree.ti,ab,hw.))

31 (risk.ti,ab,hw. AND (exp algorithm/ OR algorithm*.ti,ab,hw. OR tool*.ti,ab,hw. OR scale*.ti,ab,hw. OR rule*.ti,ab,hw. OR checklist*.ti,ab,hw. OR rating*.ti,ab,hw. OR score*.ti,ab,hw. OR index.ti,ab,hw. OR indices.ti,ab,hw. OR model*.ti,ab,hw. OR exp machine learning/ OR machine learning.ti,ab,hw. OR exp "decision tree"/ OR decision tree.ti,ab,hw.))

32 23 OR 24 OR 25 OR 26 OR 27 OR 28 OR 29 OR 30 OR 31

33 (1 OR 11) AND 22 AND 32

**PsycINFO**

**Search terms for suicide:**

1 exp Suicide/ OR Suicid*.ti,ab,hw,id.

**Search terms for self-harm:**

2 (self$harm* OR self-harm*).ti,ab,hw,id.

3 (exp Self-Injurious Behavior/ OR self$injur*.ti,ab,hw,id. OR self-injur*.ti,ab,hw,id.)

4 (self$poison* OR self-poison*).ti,ab,hw,id.

5 (para$suicid* OR para-suicid*).ti,ab,hw,id.

6 (self$mutilat*).ti,ab,hw,id.

7 (exp Drug Overdoses/ OR overdos*.ti,ab,hw,id.)

8 ((auto ADJ (aggress* OR mutilat*)) OR (autoaggress* OR automutilat*)).ti,ab,hw,id.

9 ((self OR themsel* OR oneself*) ADJ2 (harm* OR cut* OR immolat* OR inflict* OR injur* OR mutilat* OR poison* OR damag* OR destruct*)).ti,ab,hw,id.

10 2 OR 3 OR 4 OR 5 OR 6 OR 7 OR 8 OR 9

**Search terms for predictive performance measures:**

11 (exp Test Sensitivity/ OR exp Test Specificity/ OR sensitivit*.ti,ab,hw,id. OR specificit*.ti,ab,hw,id.)

12 ((predict* ADJ3 value*) OR PPV OR NPV).ti,ab,hw,id.

13 ((pre$test OR pre-test OR post$test OR post-test) ADJ2 probabilit*).ti,ab,hw,id.

14 ((pre$test OR pre-test OR post$test OR post-test) ADJ2 odd*).ti,ab,hw,id.

15 (likelihood ADJ3 ratio*).ti,ab,hw,id.

16 (Diagnostic odds ratio).ti,ab,hw,id.

17 ((c$statistic* OR C-statistic*) OR (c$index* OR C-index*) OR (d$statisic* OR D-statistic*) OR ((area under ADJ2 curve) OR AUC OR receiver operating characteristic OR (roc ADJ2 (analy* OR curv* OR plot*)))).ti,ab,hw,id.

18 (Calibrat* OR Brier score OR (Hosmer ADJ1 Lemeshow)).ti,ab,hw,id.

19 (Net reclassification improvement OR net reclassification index OR NRI).ti,ab,hw,id.

20 (Integrated discrimination improvement OR IDI).ti,ab,hw,id.

21 11 OR 12 OR 13 OR 14 OR 15 OR 16 OR 17 OR 18 OR 19 OR 20

**Search terms for risk prediction models:**

22 (exp Risk Assessment/ OR risk assessment.ti,ab,hw,id.)

23 exp Decision Support Systems/

24 exp "checklist (testing)"/ OR exp rating scales/ OR exp symptom checklists/

25 (Predict*.ti. OR Rule*.ti,ab,hw,id.)

26 (Predict* AND Model*).ti,ab,hw,id.

27 (Decision* AND (Model* OR Clinical*)).ti,ab,hw,id.

28 (Prognostic AND (Scor* OR Model*)).ti,ab,hw,id.

29 ((screen* OR assess* OR predict* OR prognos*).ti,ab,hw,id. AND (exp Algorithms/ OR algorithm*.ti,ab,hw,id. OR tool*.ti,ab,hw,id. OR scale*.ti,ab,hw,id. OR rule*.ti,ab,hw,id. OR checklist*.ti,ab,hw,id. OR rating*.ti,ab,hw,id. OR score*.ti,ab,hw,id. OR index.ti,ab,hw,id. OR indices.ti,ab,hw,id. OR model*.ti,ab,hw,id. OR exp Machine Learning Algorithms/ OR machine learning.ti,ab,hw,id. OR decision tree.ti,ab,hw,id.))

30 (risk.ti,ab,hw,id. AND (exp Algorithms/ OR algorithm*.ti,ab,hw,id. OR tool*.ti,ab,hw,id. OR scale*.ti,ab,hw,id. OR rule*.ti,ab,hw,id. OR checklist*.ti,ab,hw,id. OR rating*.ti,ab,hw,id. OR score*.ti,ab,hw,id. OR index.ti,ab,hw,id. OR indices.ti,ab,hw,id. OR model*.ti,ab,hw,id. OR exp Machine Learning Algorithms/ OR machine learning.ti,ab,hw,id. OR decision tree.ti,ab,hw,id.))

31 22 OR 23 OR 24 OR 25 OR 26 OR 27 OR 28 OR 29 OR 30

32 (1 OR 10) AND 21 AND 31

**CINAHL**

**Search terms for suicide:**

1 (MH "Suicide+") OR TX Suicid*

**Search terms for self-harm:**

2 (TX (self W1 harm*))

3 ((MH "Self-Injurious Behavior") OR (MH "Injuries, Self-Inflicted") OR TX (self W1 injur*))

4 (TX (self W1 poison*))

5 (TX (parasuicid* OR para W1 suicid*))

6 (TX (self W1 mutilat*))
7 ((MH "Overdose+") OR TX overdos*)

8 (TX ((auto W1 (aggress* OR mutilat*)) OR autoaggress* OR automutilat*))

9 (TX ((self OR themsel* OR oneself*) N2 (harm* OR cut* OR immolat* OR inflict* OR injur* OR mutilat* OR poison* OR damag* OR destruct*)))

10 2 OR 3 OR 4 OR 5 OR 6 OR 7 OR 8 OR 9

**Search terms for predictive performance measures:**

11 ((MH "Sensitivity and Specificity") OR TX (sensitivit* OR specificit*))

12 ((MH "Predictive Value of Tests") OR TX ((predict* N3 value*) OR PPV OR NPV))

13 (TX ((pretest OR "pre-test" OR posttest OR "post-test") N2 probabilit*))

14 (TX ((pretest OR "pre-test" OR posttest OR "post-test) N2 odd*))

15 (TX (likelihood N3 ratio*))

16 (TX “diagnostic odds ratio”)

17 ((MH "ROC Curve") OR TX (“C-statistic” OR “C-index” OR “D-statistic” OR ((“area under” N2 curve) OR AUC OR “receiver operating characteristic” OR (roc N2 (analy* OR curv* OR plot*)))))

18 (TX (Calibrat* OR “Brier score” OR (Hosmer N1 Lemeshow)))

19 (TX (“Net reclassification improvement” OR “net reclassification index” OR NRI)

20 (“Integrated discrimination improvement” OR IDI))

21 11 OR 12 OR 13 OR 14 OR 15 OR 16 OR 17 OR 18 OR 19 OR 20

**Search terms for risk prediction models:**

22 ((MH "Risk Assessment") OR TX “risk assessment”)

23 ((MH "Decision Support Techniques+") OR (MH "Decision Support Systems, Clinical") OR (MH "Decision Support Systems, Management"))

24 (MH "Checklists")

25 (TX (Predict* OR Rule*))

26 (TX (Predict* AND Model*))

27 (TX (Decision* AND (Model* OR Clinical*)))

28 (TX (Prognostic AND (Scor* OR Model*)))

29 (TX (screen* OR assess* OR predict* OR prognos*) AND ((MH "Algorithms") OR TX (algorithm* OR tool* OR scale* OR rule* OR checklist* OR rating* OR score* OR index OR indices OR model* OR machine learning OR decision tree)))

30 (TX risk AND ((MH "Algorithms") OR TX (algorithm* OR tool* OR scale* OR rule* OR checklist* OR rating* OR score* OR index OR indices OR model* OR “machine learning” OR “decision tree”)))

31 22 OR 23 OR 24 OR 25 OR 26 OR 27 OR 28 OR 29 OR 30

32 (1 OR 10) AND 21 AND 31

**Global Health**

**Search terms for suicide:**
1 exp suicide/ OR Suicid*.ti,ab,hw.

**Search terms for self-harm:**

2 (self$harm* OR self-harm*).ti,ab,hw.

3 (self$injur* OR self-injur*).ti,ab,hw.

4 (self$poison* OR self-poison*).ti,ab,hw.

5 (para$suicid* OR para-suicid*).ti,ab,hw.

6 self$mutilat*.ti,ab,hw.

7 (exp overdose/ OR overdos*.ti,ab,hw.)

8 ((auto ADJ (aggress* OR mutilat*)) OR (autoaggress* OR automutilat*)).ti,ab,hw.

9 ((self OR themsel* OR oneself*) ADJ2 (harm* OR cut* OR immolat* OR inflict* OR injur* OR mutilat* OR poison* OR damag* OR destruct*)).ti,ab,hw.

10 2 OR 3 OR 4 OR 5 OR 6 OR 7 OR 8 OR 9

**Search terms for predictive performance measures:**

11 (sensitivit* OR specificit*).ti,ab,hw.

12 ((predict* ADJ3 value*) OR PPV OR NPV) .ti,ab,hw.

13 ((pre$test OR pre-test OR post$test OR post-test) ADJ2 probabilit*).ti,ab,hw.

14 ((pre$test OR pre-test OR post$test OR post-test) ADJ2 odd*).ti,ab,hw.

15 (likelihood ADJ3 ratio*).ti,ab,hw.

16 (Diagnostic odds ratio) .ti,ab,hw.

17 ((c$statistic* OR C-statistic*) OR (c$index* OR C-index*) OR (d$statisic* OR D-statistic*) OR ((area under ADJ2 curve) OR AUC OR receiver operating characteristic OR (roc ADJ2 (analy* OR curv* OR plot*)))).ti,ab,hw.

18 (Calibrat* OR Brier score OR (Hosmer ADJ1 Lemeshow)) .ti,ab,hw.

19 (Net reclassification improvement OR net reclassification index OR NRI).ti,ab,hw.

20 (Integrated discrimination improvement OR IDI).ti,ab,hw.

21 11 OR 12 OR 13 OR 14 OR 15 OR 16 OR 17 OR 18 OR 19 OR 20

**Search terms for risk prediction models:**

22 (exp risk assessment/ OR risk assessment.ti,ab,hw.)

23 exp checklists/

24 (Predict*.ti. OR Rule*.ti,ab,hw.)

25 (Predict* AND Model*).ti,ab,hw.

26 (Decision* AND (Model* OR Clinical*)).ti,ab,hw.

27 (Prognostic AND (Scor* OR Model*)).ti,ab,hw.

28 ((screen* OR assess* OR predict* OR prognos*).ti,ab,hw. AND (exp algorithms/ OR (algorithm* OR tool* OR scale* OR rule* OR checklist* OR rating* OR score* OR index OR indices OR model* OR machine learning OR decision tree).ti,ab,hw.))

29 (risk.ti,ab,hw. AND (exp algorithms/ OR (algorithm* OR tool* OR scale* OR rule* OR checklist* OR rating* OR score* OR index OR indices OR model* OR machine learning OR decision tree).ti,ab,hw.))

30 22 OR 23 OR 24 OR 25 OR 26 OR 27 OR 28 OR 29

32 (1 OR 10) AND 21 AND 30

## List of data extraction items

**Key study characteristics**

- Type of prediction modelling study (development, external validation, combined development and external validation)
- Number of developed and/or validated models
- Validated model (if applicable)
- Type of external validation (if applicable)
- Country
- Data source
- Study dates
- Setting
- Target population
- Study design

**Participants**

- Recruitment strategy
- Number of centres
- Inclusion and exclusion criteria
- Age: mean (standard deviation) or median (interquartile range)
- Sex: number and proportion of females

**Predictors**

- Number of candidate predictor parameters
- Number of predictors included in the final model
- Predictors included in the final model

**Outcome**

- Outcome definition
- Outcome measurement method
- Intended timing of model use for prediction
- Prediction horizon
- Follow-up duration: mean (standard deviation) or median (interquartile range)

**Sample size**

- Total number of participants and total number of participants with the outcome (model development)
- Number of events per candidate predictor parameter
- Total number of participants and total number of participants with the outcome in the testing set (if a split-sample approach was used for internal validation)
- Total number of participants and total number of participants with the outcome (external validation)

**Analysis methods**

- Reporting of the amount of missing data
- Method of handling missing data
- Model development method
- Method of variable selection prior to multivariable modelling
- Method of variable selection during multivariable modelling
- Whether internal validation was performed
- Internal validation technique (if applicable)
- Shrinkage method (if applicable)
- Use of class imbalance correction methods
- Clustering (multiple observations per person included in the analysis)

**Model performance (recorded separately for apparent performance, internal validation, and external validation)**

- Calibration (calibration plot, calibration slope, calibration-in-the-large, expected to observed ratio, whether the Hosmer-Lemeshow test was used to assess calibration)
- Discrimination (C index and confidence interval)
- List of classification measures reported
- Net benefit

**Model presentation**

- Presentation of the model as a regression equation or a tool (e.g. a nomogram or online calculator)

## Additional details on data extraction and risk of bias assessment

For combined model development and external validation articles, we extracted data and assessed the risk of bias separately for the development and validation components.

Where the sample size for model development and/or number of candidate predictor parameters was not fully reported, we extracted whatever information was available to enable calculation of events per candidate predictor parameter (EPP) or its range (e.g. if the exact number of predictors or events was unknown but it was clear that the EPP was smaller than 10, we recorded this). For articles that used a random split-sample approach but did not report sample size information for the training and test sets separately, we approximated the number of participants and events for model development and validation where possible (e.g. based on the random split proportions).

# Supplementary results

## Additional details on included external validation studies

Of the 29 external validations, eight (28%) were reported in the same paper in which the model development was described, and the remaining 21 (72%) were dedicated external validations in a study other than the model development study. Seven external validations [18, 56]  were conducted by independent investigators (i.e. did not have overlapping authors with the model development study). Of the 14 externally validated models, three [54, 57] were validated five times, three [5, 54] were validated twice, and the remaining eight models [19, 20, 42, 117, 119]  were validated once.

Our search update identified two studies [21, 22]  which were described as external validations of a model developed by Simon et al [54].  However, the models fitted in these studies had a different number of predictors and/or predictor coefficients to the original model described in Simon et al [54]. These studies were not included as they cannot be considered external validations of the original Simon model.

We identified two external validations [23, 58]  of the Walsh model [57] from the same centre, both of which assessed the same cohort of patients and had overlapping time periods (the validation by Walsh [58]  included data from June 2019 to April 2020, and the validation by Wilimitis [23]  included data from June 2019 to September 2020). Given the longer study period and the larger sample size, we have only included the Wilimitis [23] validation in our review. However, we report the calibration results from the Walsh validation, as the Wilimitis study did not assess calibration.

## Additional details on number of predictors included in the final model

The final number of predictors was not reported for 20 models (12%), and another 54 models (32%) were developed using methods which did not result in a discrete set of predictors included in the final model (e.g. models developed using some machine learning approaches). Thirteen models (8%) did not present any information about the types of predictors included in the final model (or the top/most important predictors, if applicable).

## Assessment of calibration performance in model development and internal validation studies

The models showed mixed calibration performance (see Konttinen [45] and Chen [43] for examples of good calibration, and Sanderson [46] and García de la Garza [49] for poorly calibrated models). Issues with the presentation of plots made it challenging to judge the accuracy of predictions for some models (see Sanderson [46, 47] for examples of incorrect assessment of calibration). We identified three studies [24, 57, 112] in which the authors reported that calibration was assessed (using calibration plots and/or the calibration intercept and slope), but presented no results from this assessment. There were also two studies [25, 51] that presented the observed outcome risk for prespecified strata of model-predicted probabilities as calibration performance. However, neither provided any information about the absolute predicted risks for these groups.

## Risk of bias in the participants, predictors, and outcome domain

In the participants domain, twenty-two percent of models were at high risk of bias, mainly due to inappropriate inclusion and exclusion criteria or the recruitment strategy making the study participants unrepresentative of the model’s intended target population. Additionally, 8% of models and 3% of validations were at unclear risk of bias due to insufficient reporting of the recruitment strategy or eligibility criteria.

Risk of bias in the predictors domain was high for 32% of models and 3% of validations, with the primary cause being unavailability of some of the predictors at the intended time of model use for prediction. For 9% of models and 3% of validations, the reporting was not sufficiently clear to assess whether the predictors were available at the models’ intended time of use, whether they were assessed in a similar way for all participants, or whether predictor measurements were influenced by knowledge of outcome data.

Almost half of developed models (48%) and 7% of validations were at unclear risk of bias in the outcome domain. This was mainly due to lack of information on the method of outcome ascertainment, the time interval between predictor measurement and outcome determination being unclear (e.g. studies without a clearly defined prediction horizon where participants had different follow-up times), or insufficient reporting on whether the outcome assessment was influenced by knowledge of predictor information. Another 19% of models were rated at high risk of bias on this domain, for example, because outcomes were assessed using a suboptimal method with high levels of measurement error, not determined in a similar way for all participants, or because the time interval between predictor assessment and outcome determination was inappropriate (e.g. studies which used predictor values measured up to the time of event to make predictions).

## Study assessing the impact of clustering on the performance of a prediction model for suicide attempts following outpatient mental health visits

Coley et al. [117] examined different approaches to accommodating clustering in terms of how the sample is divided into training and test sets (person- or visit-level) and how the training set is divided for 10-fold cross-validation to select tuning parameters (person- or visit-level). They found that using a person-level (rather than visit-level) split for dividing clustered data into training and test sets and for cross-validation led to less bias in estimates of model predictive performance. They also found that using within-cluster resampling for model estimation (to limit the impact of informative cluster size on predictive performance) provided only marginal benefits compared to an observed cluster analysis approach, and concluded that these benefits were not substantial enough to justify the use of this approach. While providing a preliminary assessment of the role of clustering in the development of prediction models, these analyses were based on a split-sample approach for model training and testing, which is not recommended [74, 75]. Furthermore, the study did not evaluate the impact of the different modelling approaches on calibration.

**Table S1 | Countries from which data were used for model development (N = 167) and external validation (N = 29)**

| **Model development** | | **External validation** | |
| --- | --- | --- | --- |
| **Country** | **N (%)** | **Country** | **N (%)** |
| USA | 99 (59%) | USA | 26 (90%) |
| Canada | 10 (6%) | Sweden | 2 (7%) |
| Denmark | 10 (6%) | Finland | 1 (3%) |
| Australia | 8 (5%) |  |  |
| England | 7 (4%) |  |  |
| Sweden | 6 (4%) |  |  |
| South Korea | 5 (3%) |  |  |
| China | 4 (2%) |  |  |
| Germany | 4 (2%) |  |  |
| Netherlands | 2 (1%) |  |  |
| Hong Kong | 1 (1%) |  |  |
| Ireland | 1 (1%) |  |  |
| Italy | 1 (1%) |  |  |
| Saudi Arabia | 1 (1%) |  |  |
| Scotland | 1 (1%) |  |  |
| Taiwan | 1 (1%) |  |  |
| Wales | 1 (1%) |  |  |
| Not specified | 5 (3%) |  |  |

**Table S2 | Additional characteristics of included models and external validations (N = 196)**

|  | **N (%)** |
| --- | --- |
| **Study designs** |  |
| Retrospective cohort using registry data (e.g. administrative or routine care hospital databases) | 79 (40%) |
| Prospective cohort | 69 (35%) |
| Nested case-control | 33 (17%) |
| Nested case-cohort | 10 (5%) |
| Randomised trial | 2 (1%) |
| Non-randomised trial | 1 (1%) |
| Other | 2 (1%) |
| **Number of centres** |  |
| Multicentre | 69 (35%) |
| Nationwide | 49 (25%) |
| Monocentre | 43 (22%) |
| Statewide | 14 (7%) |
| Citywide | 9 (5%) |
| Other | 5 (3%) |
| Not reported | 7 (4%) |
| **Outcome measurement methods** |  |
| ICD codes | 83 (42%) |
| Combined | 29 (15%) |
| Structured clinical interview | 24 (12%) |
| Self-reported questionnaire | 10 (5%) |
| Other diagnostic codes | 1 (1%) |
| Other | 13 (7%) |
| Unclear | 36 (18%) |


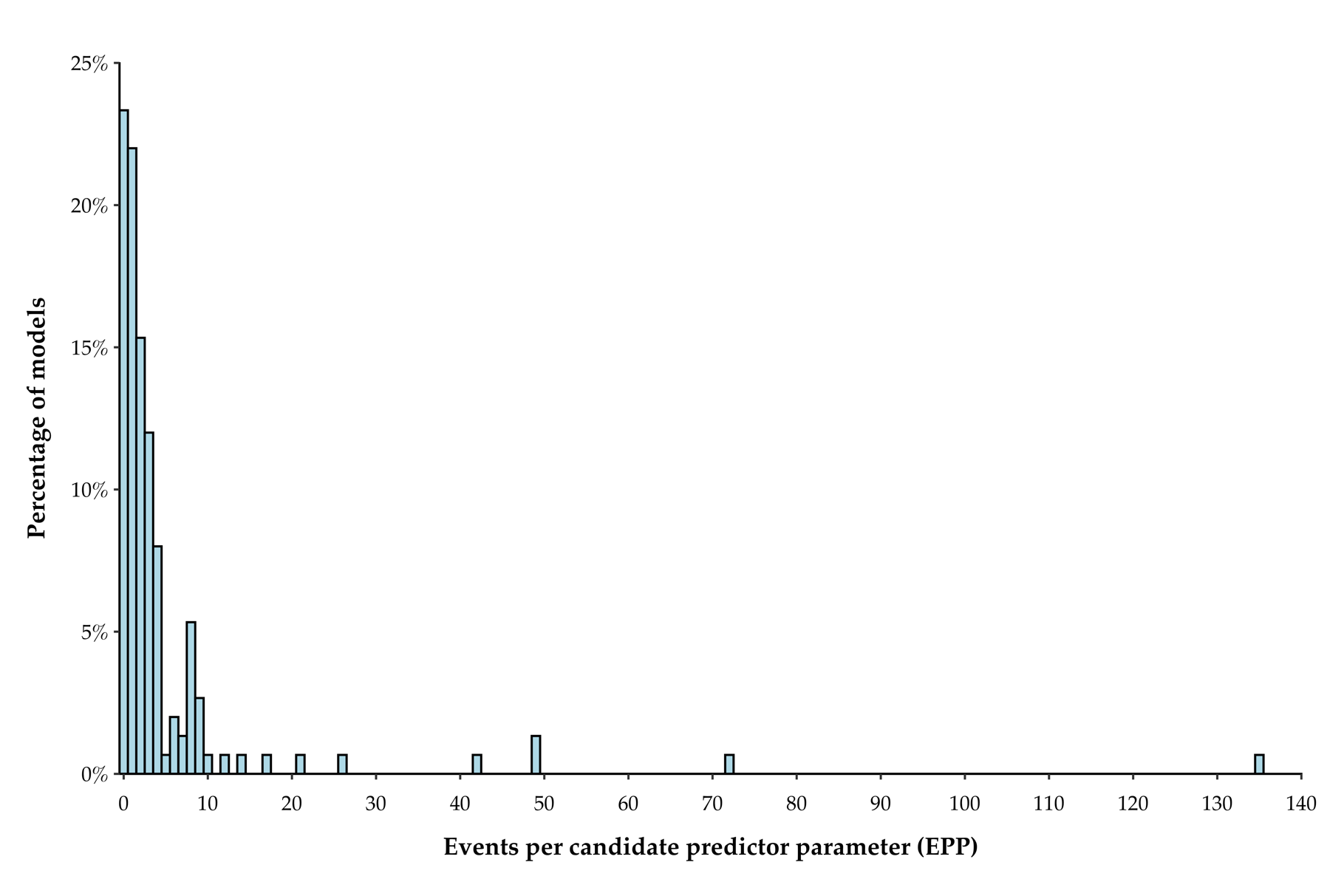


**Fig. S1 | Histogram of the number of events per candidate predictor parameter (EPP) based on the 150 models for which EPP could be calculated or approximated**


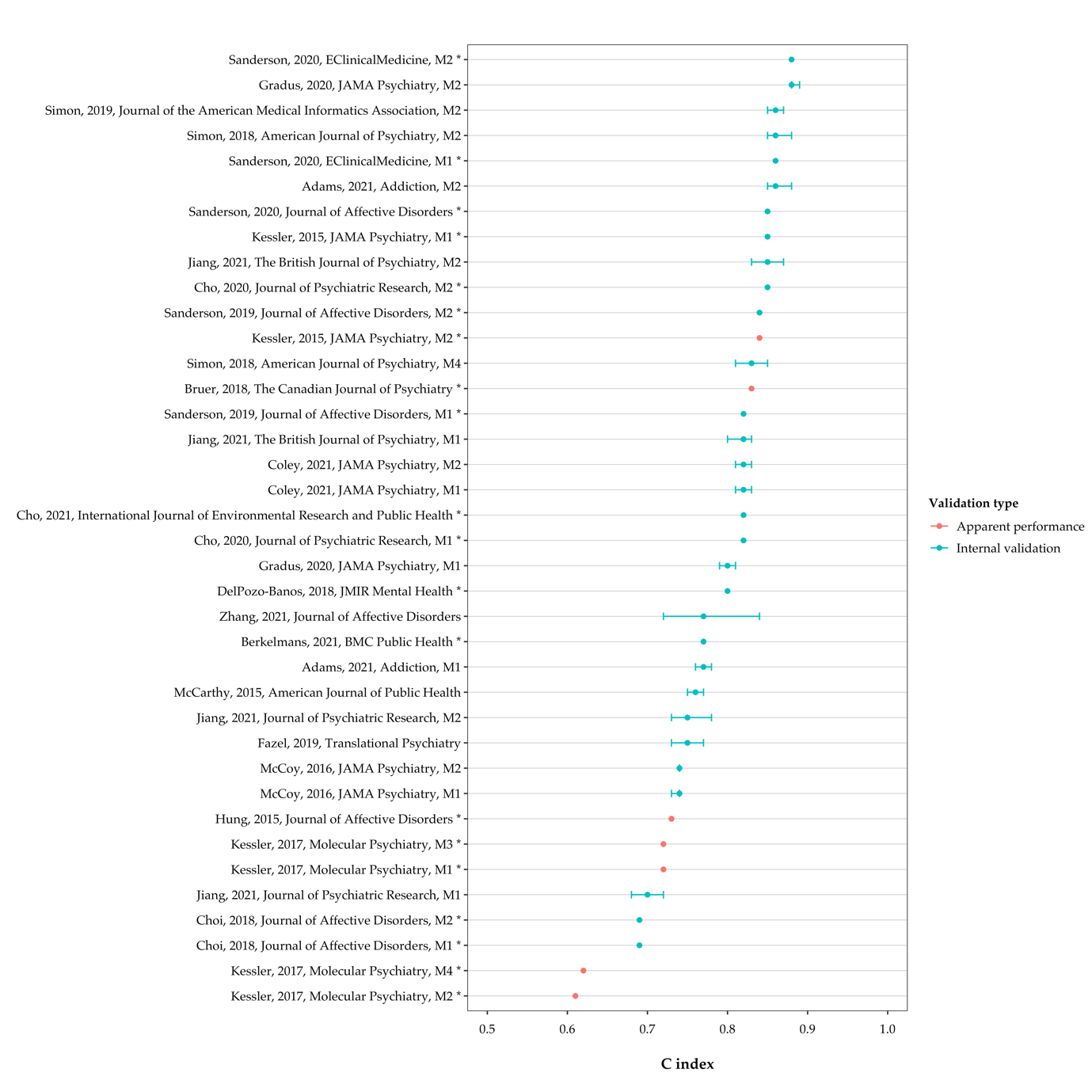


**Fig. S2 | C indexes and 95% confidence intervals of suicide death models from development studies.** Each row represents the C index reported in the development study for the corresponding model on the Y-axis (see Additional file 4 for descriptions of the models). Estimates are from internal validation where available (i.e. apparent performance results are only presented for models which were not internally validated). Confidence intervals were not reported for models marked with an asterisk. Error bars around some point estimates are not visible due to the confidence interval being too narrow.


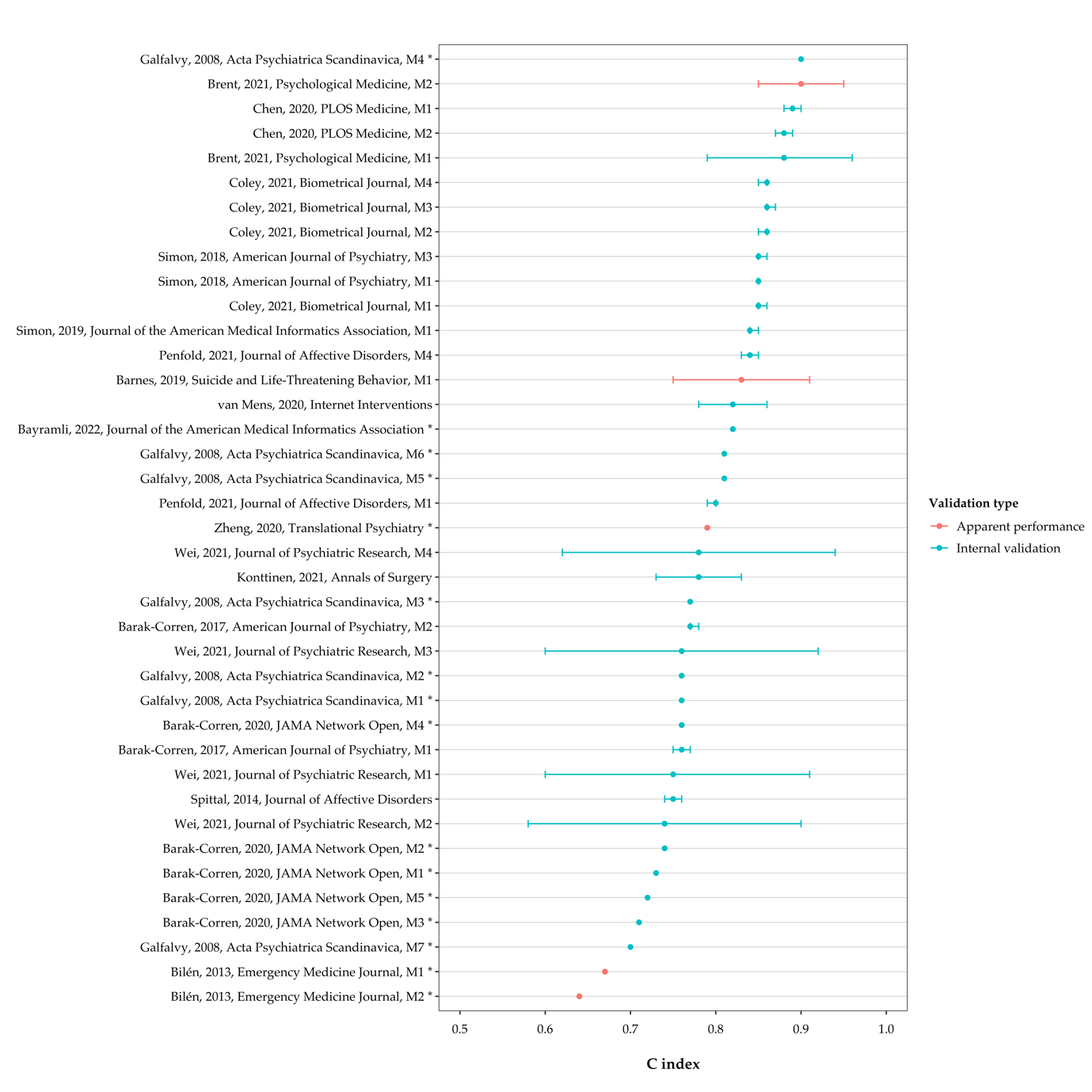


**Fig. S3 | C indexes and 95% confidence intervals of composite outcome models from development studies.** Each row represents the C index reported in the development study for the corresponding model on the Y-axis (see Additional file 4 for descriptions of the models). Estimates are from internal validation where available (i.e. apparent performance results are only presented for models which were not internally validated). Confidence intervals were not reported for models marked with an asterisk. Error bars around some point estimates are not visible due to the confidence interval being too narrow.


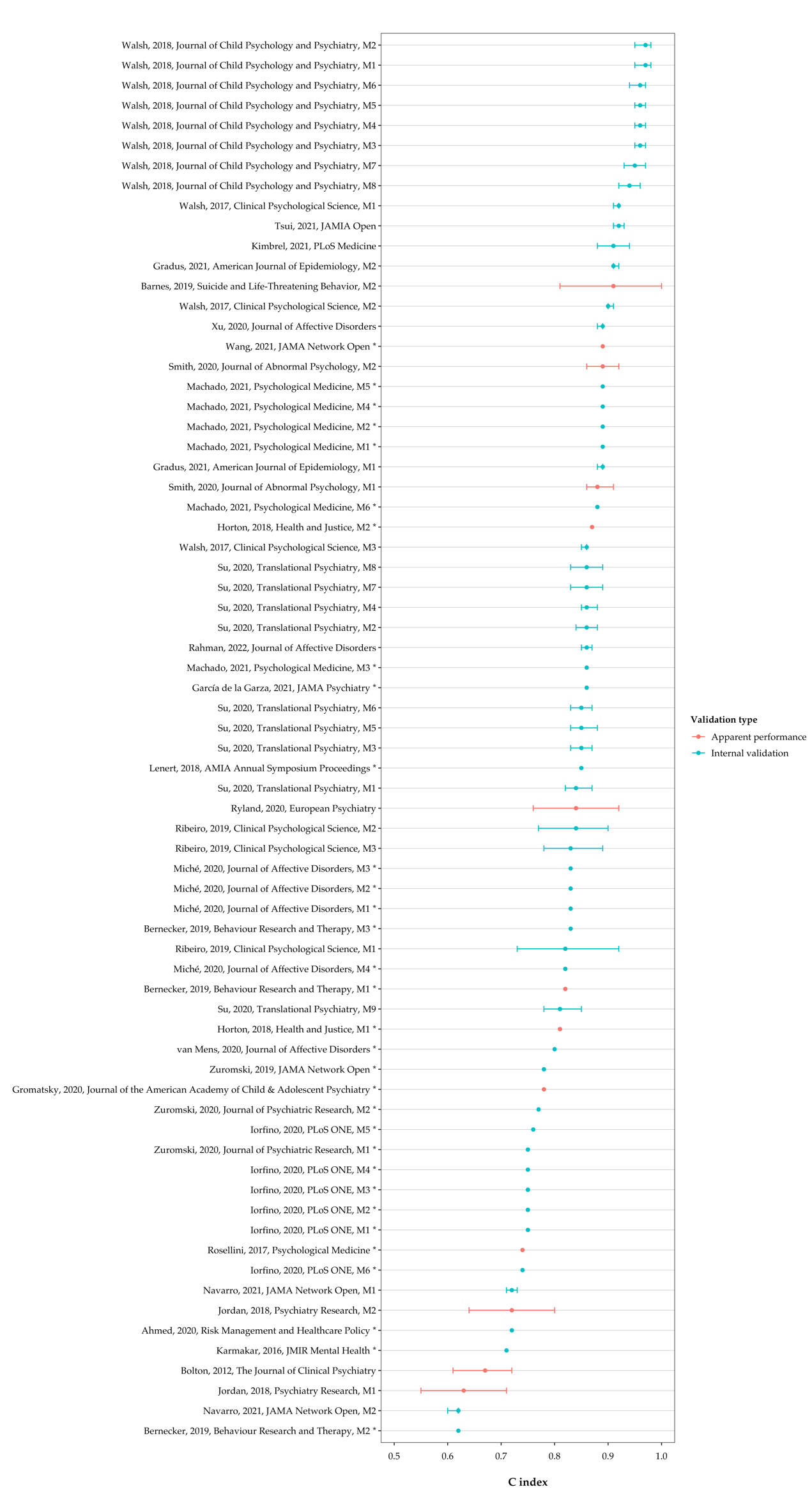


**Fig. S4 | C indexes and 95% confidence intervals of non-fatal self-harm models from development studies.** Each row represents the C index reported in the development study for the corresponding model on the Y-axis (see Additional file 4 for descriptions of the models). Estimates are from internal validation where available (i.e. apparent performance results are only presented for models which were not internally validated). Confidence intervals were not reported for models marked with an asterisk. Error bars around some point estimates are not visible due to the confidence interval being too narrow.
